# Myelin insulation as a risk factor for axonal degeneration in autoimmune demyelinating disease

**DOI:** 10.1038/s41593-023-01366-9

**Published:** 2023-06-29

**Authors:** Erik Schäffner, Mar Bosch-Queralt, Julia M. Edgar, Maria Lehning, Judith Strauß, Niko Fleischer, Theresa Kungl, Peter Wieghofer, Stefan A. Berghoff, Tilo Reinert, Martin Krueger, Markus Morawski, Wiebke Möbius, Alonso Barrantes-Freer, Jens Stieler, Ting Sun, Gesine Saher, Markus H. Schwab, Christoph Wrede, Maximilian Frosch, Marco Prinz, Daniel S. Reich, Alexander Flügel, Christine Stadelmann, Robert Fledrich, Klaus-Armin Nave, Ruth M. Stassart

**Affiliations:** 1 Department of Neurogenetics, Max Planck Institute for Multidisciplinary Sciences, Göttingen, Germany; 2 Paul Flechsig Institute of Neuropathology, University Clinic Leipzig, Leipzig, Germany; 3 Institute of Infection, Immunity and Inflammation, College of Medical Veterinary and Life Sciences, University of Glasgow, Glasgow, UK; 4 Institute of Neuroimmunology and Multiple Sclerosis Research, University Medical Center Göttingen, Göttingen, Germany; 5 Institute of Anatomy, Leipzig University, Leipzig, Germany; 6 Cellular Neuroanatomy, Institute of Theoretical Medicine, Medical Faculty, University of Augsburg, Augsburg, Germany; 7 German Center for Neurodegenerative Diseases (DZNE), Munich, Germany; 8 Department of Neurophysics, Max Planck Institute for Human Cognitive and Brain Sciences, Leipzig, Germany; 9 Paul Flechsig Institute of Brain Research, Leipzig University, Leipzig, Germany; 10 Institute of Functional and Applied Anatomy, Research Core Unit Electron Microscopy, Hannover Medical School, Hannover, Germany; 11 Institute of Neuropathology, Medical Faculty, University of Freiburg, Freiburg, Germany; 12 Centre for NeuroModulation (NeuroModBasics), University of Freiburg, Freiburg, Germany; 13 Signalling Research Centres BIOSS and CIBSS, University of Freiburg, Freiburg, Germany; 14 Translational Neuroradiology Section, National Institute of Neurological Disorders and Stroke, National Institutes of Health, Bethesda, MD USA; 15 Institute of Neuropathology, University Medical Center Göttingen, Göttingen, Germany

**Keywords:** Multiple sclerosis, Oligodendrocyte, Multiple sclerosis

## Abstract

Axonal degeneration determines the clinical outcome of multiple sclerosis and is thought to result from exposure of denuded axons to immune-mediated damage. Therefore, myelin is widely considered to be a protective structure for axons in multiple sclerosis. Myelinated axons also depend on oligodendrocytes, which provide metabolic and structural support to the axonal compartment. Given that axonal pathology in multiple sclerosis is already visible at early disease stages, before overt demyelination, we reasoned that autoimmune inflammation may disrupt oligodendroglial support mechanisms and hence primarily affect axons insulated by myelin. Here, we studied axonal pathology as a function of myelination in human multiple sclerosis and mouse models of autoimmune encephalomyelitis with genetically altered myelination. We demonstrate that myelin ensheathment itself becomes detrimental for axonal survival and increases the risk of axons degenerating in an autoimmune environment. This challenges the view of myelin as a solely protective structure and suggests that axonal dependence on oligodendroglial support can become fatal when myelin is under inflammatory attack.

## Abstract (web summary)

Myelin is widely believed to protect axons from damage in multiple sclerosis. The authors challenge this view and find that myelin insulation renders axons more vulnerable to degeneration in an autoimmune environment.

## Main

Multiple sclerosis is an inflammatory, demyelinating neurological disorder of the central nervous system (CNS) and the most common cause of sustained disability in young adults. Axonal degeneration defines the progression of clinical symptoms in affected patients^1^ but the determinants of axonal vulnerability for irreversible damage remain poorly understood.

Demyelination is widely considered to be the principal cause of axon degeneration in multiple sclerosis lesions. However, axonal pathology is an early feature in human multiple sclerosis and experimental autoimmune encephalomyelitis (EAE), with focal axonal damage observable within hours after EAE onset, and hence before the overt loss of myelin^2–7^. Indeed, axonal transport deficits as well as local rises in axoplasmic calcium have been observed even in axons with still intact-appearing myelin sheaths in acute neuroinflammatory lesions^6,7^. Moreover, oligodendrocyte death is not a prominent feature of early lesions in multiple sclerosis and its models, and oligodendrocytes survive for an extended period of time under neuroinflammatory conditions^8^. Despite this, and based on the assumption that demyelinated and unmyelinated axons are especially vulnerable to injury, initial axonal damage in multiple sclerosis is proposed to occur at areas naturally devoid of myelin, such as nodes of Ranvier^5–7^. Oligodendrocytes provide more than mere electrical insulation to axons, and long-term axonal integrity critically depends on oligodendroglial health, a finding originally derived from studies in myelin mutants^9,10^. Indeed, we and others suggested that oligodendrocytes provide trophic support to axons^11–13^, because axons are isolated from extracellular glucose at least in part by myelin itself^10^. In addition, oligodendrocytes contribute to antioxidant defense and protect neurons from cytotoxicity^14^. Consequently, fully myelinated axons should be especially dependent on oligodendroglial integrity, which, in turn, may render them specifically vulnerable to autoimmune attacks that impact the supportive capacity of oligodendrocytes.

Here we sought to challenge the prevailing concept of myelin as a solely protective structure for axons in multiple sclerosis. We reasoned that oligodendrocytes, when exposed to an acute inflammatory milieu, may lose their ability to support axonal integrity, turning myelin insulation into a threat for axonal survival. Indeed, by examining axonal damage as a function of myelin in human multiple sclerosis biopsies and in standard murine models of experimental demyelination, we demonstrate as a proof of principle that in an autoimmune environment, the fate of an axon crucially depends on its myelination state. Remarkably, we found irreversible axonal damage to be restricted to myelinated axons in autoimmune lesions, even when the proportion of unmyelinated axons was artificially increased in a genetically engineered mouse model.

Together, our data support a model according to which dysfunctional myelinating oligodendrocytes fail to sustain axonal support in an acute inflammatory environment, with detrimental consequences for axonal survival.

## Results

### Two main types of axonal pathology in human multiple sclerosis lesions

**Fig. 1:**
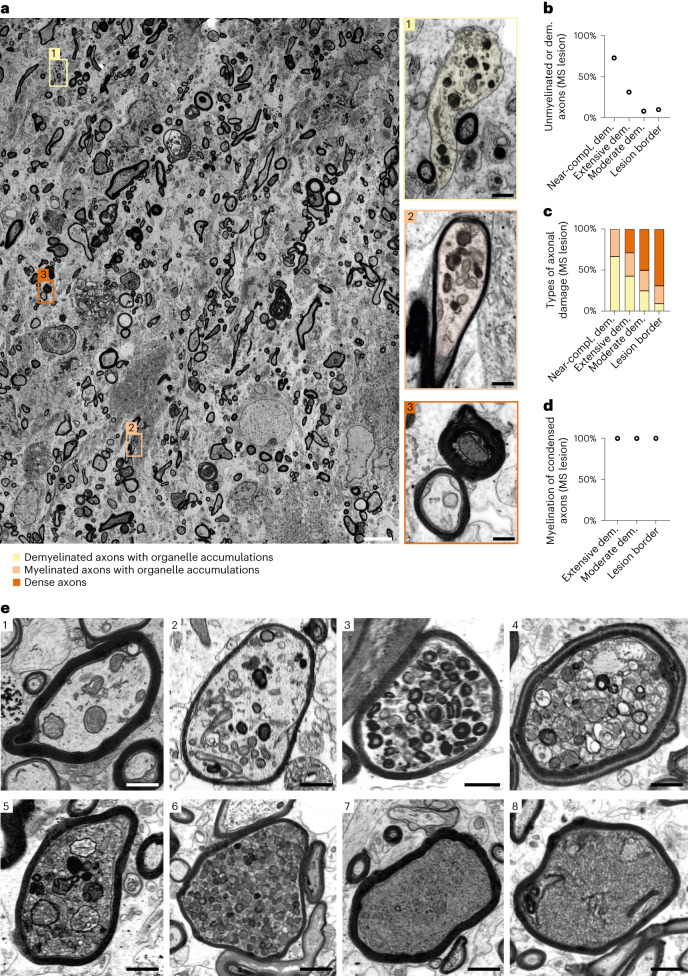
In human MS lesions, irreversible axonal damage prevails in myelinated fibers. **a**, Representative electron micrograph of a human multiple sclerosis (MS) lesion with moderate demyelination activity. Common types of axonal damage are exemplified, including demyelinated axons with organelle accumulation (box 1, yellow), myelinated axons with organelle accumulation (box 2, light orange) and axons with condensed axoplasm (box 3, dark orange). Scale bars, 5 µm; 0.5 µm in boxes 1–3. **b**, Demyelinated and unmyelinated axons (as a percentage of all axons) in four lesions from individual human MS lesions (lesion with near-complete demyelination, active lesion with extensive demyelination, active lesion with moderate demyelination and lesion border with limited demyelination). Each point represents one lesion. **c**, The damage types (listed above) show an inverse correlation between the extent of demyelination in the four MS lesions and the percentage of irreversibly damaged axons (dense axons). **d**, The myelination status of condensed axons reveals that irreversibly damaged axons are always myelinated, independent of the lesion characteristics. Each point represents one lesion. **e**, Electron microscopic images of axonal pathology in human MS samples demonstrating the heterogeneity of axonal organelle accumulations and axons with highly condensed axoplasm, as well as potential transition types between both forms of axonal damage from 1 to 8. Scale bars, 1 µm.

**Extended Data Fig. 1:**
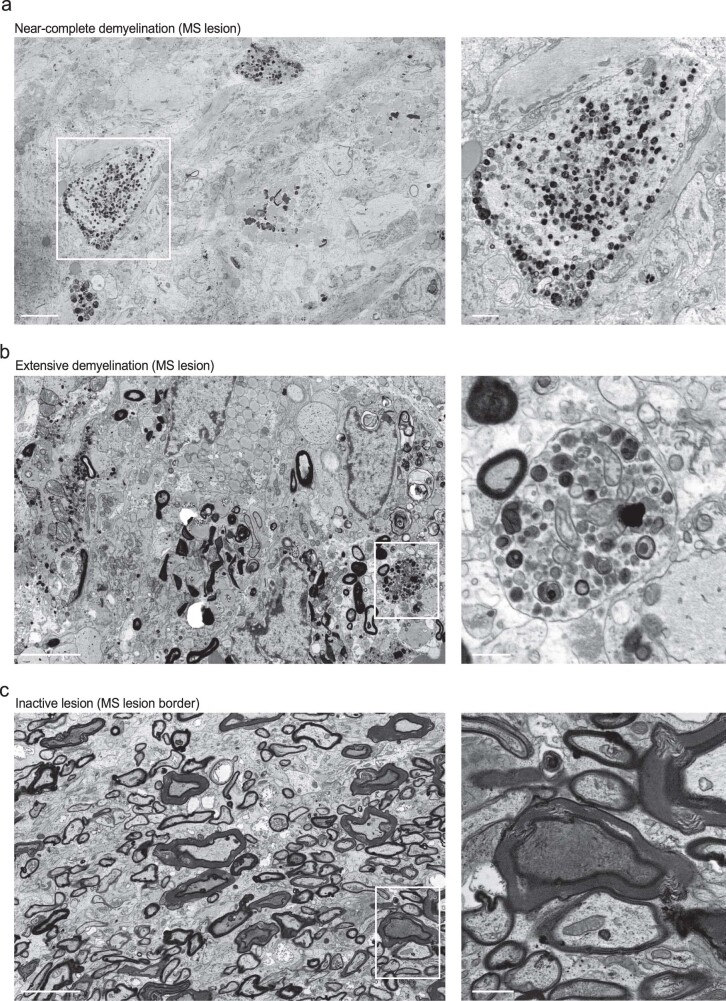
Human multiple sclerosis lesions. **a**, Representative electron micrograph of a lesion with near-complete demyelination. Note the virtual absence of myelinated axons. A demyelinated axon with organelle accumulation is shown in higher magnification. Scale bar = 5µm / blow-up = 1µm. **b**, A representative image from an active lesion with extensive demyelination demonstrates a few myelinated axons, ongoing phagocytosis and many demyelinated axons. A demyelinated axon with organelle accumulation is shown at higher magnification. Scale bar = 5µm / blow-up = 1µm. **c**, A representative image of a lesion border with a large proportion of myelinated axons. Note the myelinated axons with a pronounced condensation of the axoplasm, representing irreversible axon damage (high magnification image). Scale bar = 5µm / blow-up = 1µm.

We first sought to reassess the relationship between demyelination and axonal damage in human multiple sclerosis, as only sparse data on the ultrastructural characteristics of axonal pathology in multiple sclerosis lesions are available. To this end, we performed electron microscopy on *n* = 4 multiple sclerosis biopsies. We analyzed three acute demyelinating lesion centers, which are characterized by mild to severe demyelination and inflammation, as well as one lesion border with only limited active inflammation (Fig. 1a–d and Extended Data Fig. 1a–c). In all lesion types, axonal pathology could be assigned to one of two categories: axons with intra-axonal organelle accumulations, and axons with highly condensed axoplasm (Fig. 1a, Extended Data Fig. 1a–c and Supplementary Fig. 1). Axonal organelle accumulations were previously observed in myelin mutant mice and shown to correlate with axonal transport deficits, secondary to a loss of oligodendroglial support of myelinated axons^9,15–17^. In addition, early signs of impaired axonal transport were also described in EAE^5,6^. While we found axonal organelle accumulations relatively more frequently in extensively demyelinating lesions, axons with highly condensed cytoplasm showed an inverse correlation, being relatively more abundant in lesions with less pronounced demyelination (Fig. 1b,c). Notably, when assessing the myelination status of the two axonal pathologies, we found all axons with condensed axoplasm to be myelinated (Fig. 1a,d and Extended Data Fig. 1c), whereas organelle accumulations were prevalent in both myelinated and demyelinated axons (Fig. 1c). Yet many completely demyelinated axons appeared normal (Fig. 1a and Supplementary Fig. 1).

Furthermore, we observed possible ‘transition’ states between both axonal pathologies, marked by organelle accumulations and condensed axoplasm, in myelinated axons (Fig. 1e).

### Temporal dynamics of axonal pathology in MS and EAE

**Fig. 2:**
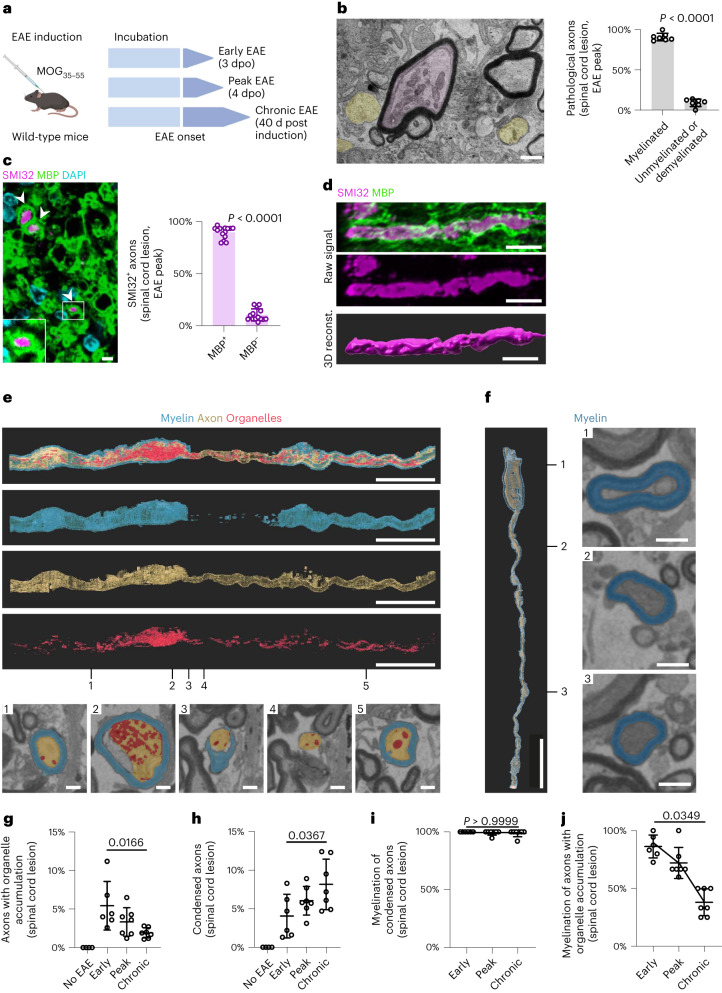
The dynamics of axonal pathology in EAE. **a**, Experimental outline. EAE was induced in wild-type mice, and spinal cord samples were collected during early, peak and chronic EAE. **b**, Electron micrograph of an EAE lesion within the ventrolateral lumbar spinal cord at 4 dpo shows a myelinated axon with organelle accumulation (pink) and intact unmyelinated axons (yellow). Scale bar, 1 µm. Quantification of pathological axons shows that most damaged axons are myelinated. Circles in the graph represent biologically independent animals (*n* = 7). **c**, Cross-section of a spinal cord lesion at 4 dpo immunostained against SMI32 (pink) and MBP (green). Arrowheads show SMI32^+^ axons with surrounding MBP^+^ myelin. Scale bar, 2.5 µm. Quantification revealed that most SMI32^+^ axons are myelinated (MBP^+^). Unpaired two-tailed Welch’s *t*-test was used in **b** and **c**. **d**, Longitudinal image and 3D reconstruction of an EAE lesion immunostained against SMI32 (pink) and MBP (green) demonstrates continuous SMI32^+^ labeling along a myelinated axon. Scale bars, 5 µm. **e**, Top, electron microscopy and 3D reconstruction of an axon with organelle accumulation that is partially covered by myelin. Scale bars, 10 µm. Bottom, electron microscopic cross-sections at indicated segments from the top panels. Scale bars, 1 µm. Organelle accumulation is predominantly found in the myelinated part of the axon. **f**, Left, electron microscopy 3D reconstruction of a myelinated condensed axon. Scale bar, 10 µm. Right, cross-sections at indicated segments from the left panel (dense axon without pseudocolor). Scale bars, 1 µm. **g**–**j**, Quantification of axon pathology in electron micrographs of spinal cord lesions during early (3 dpo), peak (4 dpo) and chronic (40 d post induction) EAE. Organelle accumulations become less frequent over time (**g**). Condensed axons accumulate over time (no pathology in healthy spinal cord, no EAE) (**h**). Most condensed axons are myelinated (**i**). Percentage of myelinated axonal swellings decreases with EAE progression (**j**). One-way ANOVA, Tukey’s multiple comparisons (**g**,**h**) or Kruskal–Wallis with Dunn’s multiple comparisons post-hoc tests (**i,j**). Exact *P* values are given for early versus chronic EAE. Circles in the graphs represent biologically independent animals (no EAE, *n* = 4; early, *n* = 6; peak and chronic, *n* = 7). Data are means ± s.d.

The highly condensed axoplasm that we observed in multiple sclerosis lesions reflects decomposing membranous structures of organelles and cytoskeletal elements (Supplementary Fig. 2a,b). This type of axonal pathology has previously been recognized as a sign of irreversible axon damage in different injury paradigms^18–23^. By contrast, axonal organelle accumulations may be potentially reversible^5^. Therefore, we hypothesized that such accumulations may represent an early consequence of autoimmune-mediated oligodendrocyte dysfunction. To determine the temporal dynamics of axonal pathologies, we induced EAE in C57/BL6 mice by immunization with myelin oligodendrocyte glycoprotein 35–55 (MOG_35–55_) (Fig. 2a and Supplementary Fig. 2c–e). In this variant of EAE, B cells and antibodies play only a minor role, and demyelination is moderate in the acute disease phase^8,24^. We first analyzed EAE lesions in the ventrolateral lumbar spinal cord at disease peak (4 days post disease onset), when immune cell infiltration is prominent throughout the lesion (Fig. 2a and Supplementary Fig. 2c–e), and assessed axonal pathology. At this early disease time point, numerous axons appeared abnormal, and we found by electron microscopy (Fig. 2b) as well as by immunohistochemistry against dephosphorylated neurofilaments (SMI32) (Fig. 2c,d and Supplementary Fig. 2f) that pathology was most prevalent in myelinated axons. Axonal loss was unlikely to account for this phenomenon at this early stage because the total number of axons per area, when corrected for immune cell occupancy, was not reduced in EAE animals compared to controls, and most phagocytosed axons harbored residual myelin sheaths (Supplementary Fig. 3a–c).

We next aimed at analysing the characteristics of injured myelinated fibers during the early phase of EAE. In addition to entire myelin–axon profiles engulfed by phagocytosing cells (Supplementary Fig. 3b,c), we detected in mouse EAE the same two types of axonal pathologies as previously seen in human multiple sclerosis: axons with organelle accumulations and axons with highly condensed axoplasm (Fig. 2b,e,f, Supplementary Figs. 2a,b and 3d,e, and Supplementary Videos 1–4). When we examined the temporal evolution of axonal pathologies in EAE, we found the number of axon cross-sections with organelle accumulations to be highest at very early time points and to subsequently gradually decrease (Fig. 2g). Conversely, the percentage of axon cross-sections with highly condensed axoplasm increased during the same time course (Fig. 2h). Importantly, almost all condensed axons were myelinated, even at 40 days post EAE induction and in areas of otherwise overt demyelination (Fig. 2i and Supplementary Fig. 3f). When we analyzed the myelin characteristics of these condensed axons, we detected a specific increase in the periaxonal cytoplasmic part of the myelin sheath (the inner tongue) compared to neighboring non-pathological myelinated axons (Supplementary Fig. 3g), an observation that has previously been made in myelin mutants and that has been suggested to reflect compromised trafficking of metabolites to the axon by damaged oligodendrocytes^9,11,16,25,26^. In line with this, oligodendroglial malfunction is also reflected at the transcriptional level in myelinating oligodendrocytes in EAE^27,28^. A re-analysis of published single-cell RNA sequencing (scRNA-seq) datasets^27,28^ revealed, for instance, a downregulation of monocarboxylate transporter 1 (MCT1; encoded by *Slc16a1*) expression, as well as a dysregulation of cellular processes related to metabolism, cell stress and the glial immune response (Supplementary Fig. 4).

**Extended Data Fig. 2:**
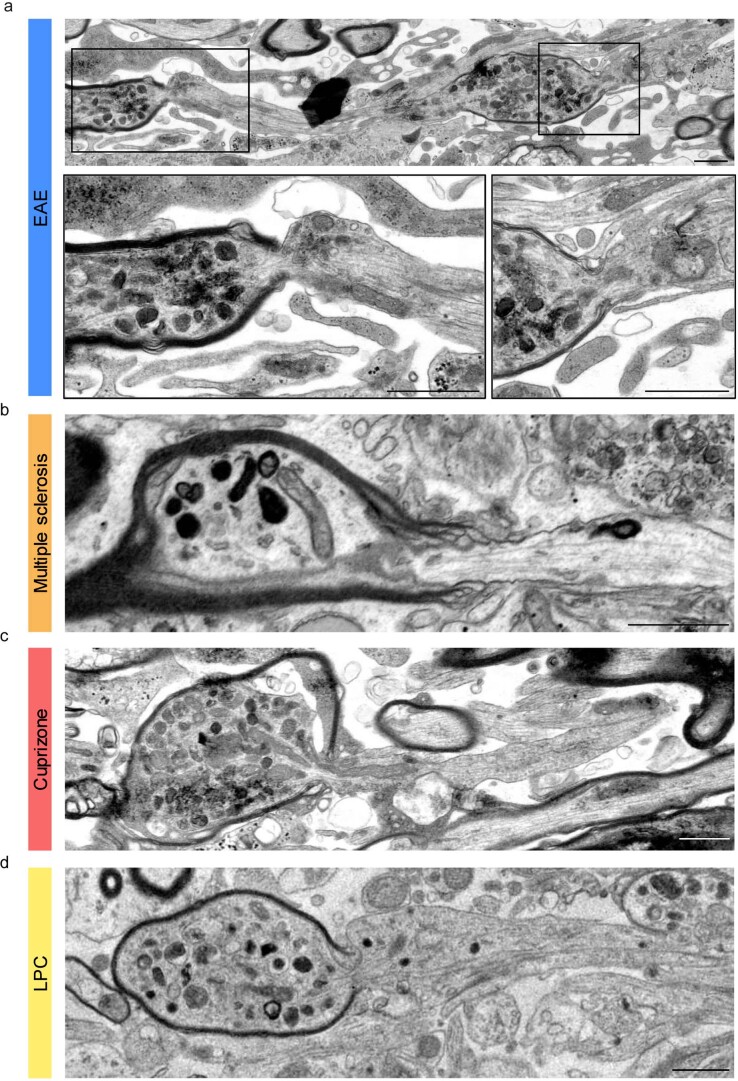
Axonal organelle accumulations predominate in myelinated segments. Images of longitudinal, partially demyelinated axons show that organelles accumulate specifically in not or not yet demyelinated parts of the axons. Similar observations were made in **a**, EAE; **b**, Multiple sclerosis; **c**, Cuprizone and **d**, Lysophosphatidylcholine (LPC) -induced demyelination. Scale bar = 3µm.

In contrast to the sustained myelination of axons with highly condensed cytoplasm, the myelination status of axons with organelle accumulations changed dynamically throughout the EAE disease course, decreasing from over 90% at 3 days post disease onset to 40% at 40 days post disease induction (Fig. 2j). Electron microscopy of individual EAE nerve fibers in 3D-reconstructed serial block-face imaging or in longitudinal sections revealed that while organelle accumulations occurred in axonal segments with remaining myelin sheaths, adjacent demyelinated segments appeared normal (Fig. 2e, Supplementary Fig. 3d, Extended Data Fig. 2a and Supplementary Videos 1–2), consistent with our findings in multiple sclerosis biopsies and in toxin-induced models of demyelination (Extended Data Fig. 2b−d).

**Fig. 3:**
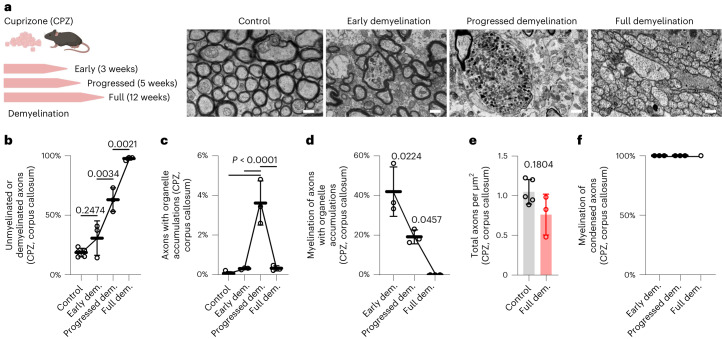
The dynamics of axonal pathology in CPZ-induced demyelination. **a**, Left, schematic outline of the experiment. Wild-type mice were fed CPZ to induce demyelination for 3 weeks (early demyelination), 5 weeks (progressed demyelination) and 12 weeks (full demyelination). The corpus callosum was analyzed. Right, representative images of the corpus callosum without CPZ treatment (Control), and with CPZ feeding for 3 weeks, 5 weeks and 12 weeks. Scale bars, 1.5 µm. **b**, The percentage of unmyelinated or demyelinated axons in CPZ lesions after different time periods of CPZ feeding. One-way ANOVA with Tukey’s multiple comparisons test (*P* values shown for comparison in chronological order). **c**, Although the percentage of axonal swellings first strongly increases along with CPZ lesion development, few axonal organelle accumulations characterize fully demyelinated CPZ lesions as revealed by quantification of electron micrographs. One-way ANOVA with Tukey’s multiple comparisons test (*P* values shown for comparison with progressed demyelination). **d**, The myelination status of axonal organelle accumulations during CPZ treatment. Axons with organelle accumulations become demyelinated over time. One-way ANOVA with Tukey’s post-hoc test. **e**, Axonal numbers in CPZ lesions show only a mild, non-significant axonal loss after 12 weeks of CPZ treatment compared to non-treated controls. Unpaired two-tailed Welch’s *t-*test. **f**, The myelination status of axons with condensed axoplasm during CPZ treatment. All condensed axons are myelinated. Note that at full demyelination, condensed axons are virtually absent and were only detected in one mouse (*n* = 1). Circles in all graphs in **b**–**f** represent independent animals (Control, *n* = 5; early demyelination, *n* = 3; progressed demyelination, *n* = 3; full demyelination, *n* = 3). Data represent means ± s.d.

**Fig. 4:**
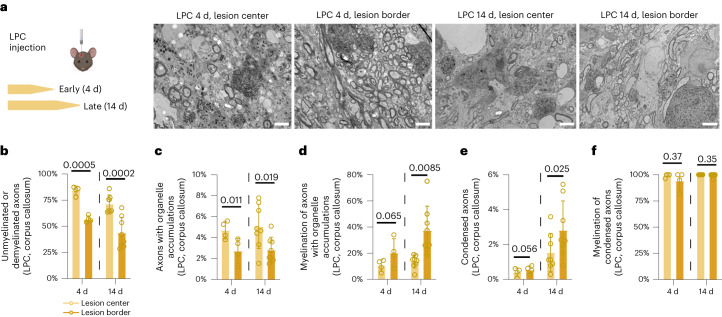
Lesion center and border differ with regard to axonal pathology in LPC-induced demyelination. **a**, Left, schematic outline of the experiment. Wild-type mice were injected with LPC in the corpus callosum to induce focal demyelination, and samples were collected at an early time point (4 d post injection) and at a late time point after demyelination (14 d post injection). Right, representative images of 4- and 14-d-old LPC lesions within the corpus callosum. **b**–**f**, Based on the extent of demyelination, lesions have been divided into efficiently demyelinated lesion centers (light yellow) and incompletely demyelinated lesion borders (dark yellow). Scale bars, 2.5 µm. **b**, Accordingly, the extent of demyelination reveals a higher percentage of demyelinated axons in the lesion center compared to the border. **c**,**e**, The percentage of axonal organelle accumulations shows a higher abundance in the lesion center (**c**), while axons with condensed axoplasm are more abundant within the lesion border (**e**). **d**,**f**, The myelination status of axonal pathology reveals that axonal organelle accumulations are a feature of both myelinated and demyelinated fibers (**d**), while condensed axons virtually always have a myelin sheath in both the lesion center and border (**f**). Unpaired two-tailed Welch’s *t-*test (**b**,**c**,**e**) or two-tailed Mann–Whitney test (**d**,**f**). Circles in all graphs represent biologically independent animals (4 d, *n* = 4; 14 d, *n* = 8). Data are shown as means ± s.d; numbers over black bars represent *P* values.

We next addressed whether complete demyelination can improve axonal integrity, resolve organelle accumulations and allow axons to escape from irreversible injury. To address this question, we took advantage of the cuprizone (CPZ) model of toxin-induced callosal demyelination in wild-type mice, in which demyelination follows a consistent time course (Fig. 3a,b). In line with our hypothesis, upon CPZ feeding, organelle-filled axons appeared and initially increased but then declined with ongoing demyelination (Fig. 3b,c). Indeed, fully demyelinated lesions contained very few residual organelle accumulations (Fig. 3c,d), supporting the concept that axonal organelle accumulations constitute an early and potentially reversible axonal injury response. Consistent with this concept, the total number of axons decreased only slightly across the time course of CPZ-induced (complete) demyelination (Fig. 3e). Notably, in these CPZ lesions, we detected a fraction of axons (4.65% of total axons) with highly condensed axoplasm, which, analogous to our findings in EAE, was a specific feature of (remaining) myelinated axons throughout the disease course (Fig. 3f and Supplementary Fig. 3f). We next postulated that if late-stage, irreversible axonal damage would indeed be the consequence of myelin retention, this type of axonal pathology should predominate in areas with incomplete demyelination compared to areas of efficient myelin loss. To address this assumption, we assessed axonal pathology in the lysophosphatidylcholine (LPC) model of focal demyelination, which allowed us to compare the efficiently demyelinated lesion center with the only partially demyelinated lesion border (Fig. 4a,b). Although we detected numerous axonal organelle accumulations at both sites (Fig. 4c,d), degeneration-prone axons with highly condensed axoplasm were relatively more abundant at the lesion border (Fig. 4e). Importantly, also in the LPC model, the condensed type of axonal pathology was restricted to myelinated fibers (Fig. 4f and Supplementary Fig. 3f).

### EAE amelioration in mutant mice with less myelination

**Fig. 5:**
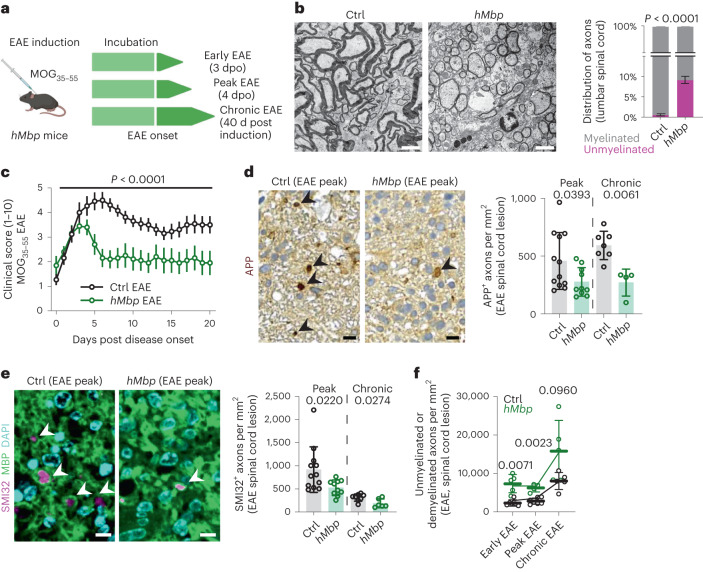
*hMbp* mice demonstrate an amelioration of the clinical disease course in EAE. **a**, Experimental outline. EAE was induced in wild-type and hypomyelinated *hMbp* mice and samples were collected during early, peak and chronic EAE. **b**, Left, representative electron micrographs of the ventrolateral spinal cord of control (Ctrl) and *hMbp* mice. Scale bars, 2.5 µm. Right, the percentage of myelinated and unmyelinated axons reveals around 10% unmyelinated fibers in the lumbar spinal cord in *hMbp* mice. Unpaired two-tailed Welch’s *t*-test; *n* = 3 biologically independent animals were analyzed. **c**, Clinical EAE scoring (1–10) shows an ameliorated disease course in *hMbp* mice compared to wild-type controls. Ctrl EAE, *n* = 13; *hMbp* EAE, *n* = 10. Two-way repeated measures ANOVA. **d**, Left, representative images of APP immunohistochemistry for axonal damage in EAE lesions. Arrowheads, APP^+^ axons. Scale bars, 10 µm. Right, the number of APP^+^ axons in EAE lesions reveals a reduced number of APP^+^ axons in *hMbp* mice at peak (4 dpo) and chronic EAE (40 d post induction) compared to wild-type EAE controls. Unpaired two-tailed Mann–Whitney test for each time point. **e**, Left, immunohistochemical images of SMI32^+^ MBP^+^ axons in EAE lesions. SMI32^+^ axons (arrowheads) are surrounded by MBP^+^ myelin sheaths. Scale bars, 5 µm. Right, quantification of SMI32^+^ axons reveals a reduced number of SMI32^+^ axons in *hMbp* mice at peak and chronic EAE compared to wild-type EAE controls. In **d** and **e**, peak Ctrl, *n* = 12; *hMbp*, *n* = 10; chronic Ctrl, *n* = 8; *hMbp*, *n* = 6. **f**, Quantification of unmyelinated or demyelinated axons in electron micrographs of EAE lesions shows that the extent of demyelination between both genotypes is similar. Note that demyelination is most prominent in the chronic EAE phase in both genotypes. Early Ctrl, *n* = 6; *hMbp*, *n* = 5; peak Ctrl, *n* = 7; *hMbp*, *n* = 4; chronic Ctrl, *n* = 5; *hMbp*, *n* = 5. Unpaired two-tailed Welch’s *t*-test per time point. Data are shown as means ± s.d. (**b**,**d**–**f**), apart from clinical scores, which are shown as means ± s.e.m. (**c**). For all graphs, numbers represent *P* values and circles represent biologically independent animals.

Collectively, our findings lead us to surmise that, contrary to general belief, persistent myelin ensheathment in the context of demyelination does not provide axonal protection, but conversely, poses a risk for axon survival. Hence, we next asked whether unmyelinated and demyelinated axons are relatively protected from axonal damage in an acute autoimmune demyelinating setting. To directly test this question, we took advantage of a new mouse mutant with reduced expression of myelin basic protein (MBP), termed hypomorphic *Mbp* (*hMbp*) mouse^29^ (Fig. 5a and Supplementary Fig. 5a,b). These mice exhibit an increased fraction of unmyelinated axons throughout the CNS, whereas the number and size of all axons appear unaffected (Fig. 5b and Supplementary Fig. 5c,d). Specifically, in the lumbar spinal cord, *hMbp* mice demonstrate around 10% of unmyelinated, predominantly medium-sized axons, compared to less than 1% in controls (Fig. 5b and Supplementary Fig. 5d,e). Remarkably, upon EAE induction, *hMbp* mutants showed a less severe clinical course of disease compared to respective wild-type controls (Fig. 5c). Moreover, axonal damage was significantly lower in EAE lesions of *hMbp* mice compared to wild-type controls at the acute and chronic disease phases, when assessed by immunohistochemistry against APP (amyloid precursor protein) and dephosphorylated neurofilament (SMI32) (Fig. 5d,e). Importantly, we observed no differences in lesion numbers, lesion sizes or localization in *hMbp* mutants compared to controls (Supplementary Fig. 5f–h). Moreover, the extent of demyelination was similar between both groups and throughout the disease course (Fig. 5f). Of note, we also did not detect differences in neuronal cell body numbers of the respective axonal fiber tracts that cross the spinal cord lesion area (Supplementary Fig. 5i).

**Fig. 6:**
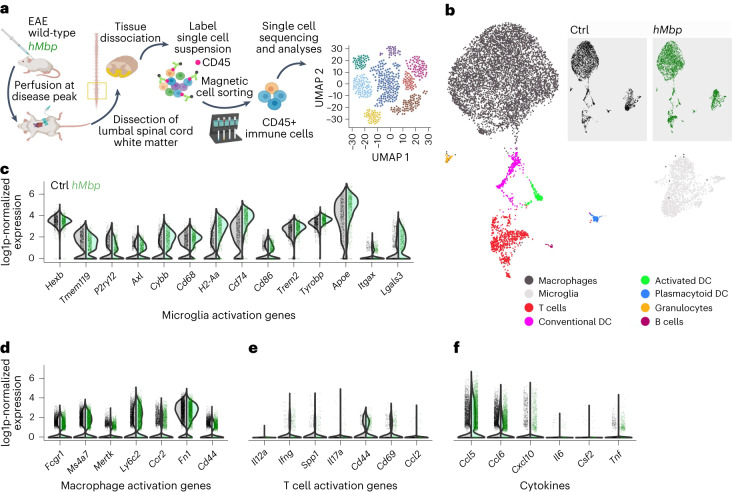
Characterization of the immune response in *hMbp* mice in response to EAE. **a**, Schematic outline of the experiment. EAE was induced in wild-type and *hMbp* mice. The ventrolateral white matter of the spinal cord was dissected at disease peak and processed to obtain a single-cell suspension, which was then labeled with magnetically labeled CD45 antibodies to specifically sort immune cells via magnetic cell sorting. Immune cells were finally processed for single-cell sequencing analyses. **b**, UMAP plot for 8,954 cells from two *hMbp* and two wild-type mice at 4 dpo. Cells are colored and annotated by cell type. Inset, UMAP plot split and colored by genotype. DC, dendritic cell. **c**–**f**, Gene expression of several marker genes for homeostatic and activated microglia (**c**), activated macrophages (**d**), activated T cells (**e**) and major cytokines (**f**) in the microglia population. No differences were observed between the genotypes. Each point represents one cell. Data are displayed as violin plots highlighting probability density.

**Extended Data Fig. 3:**
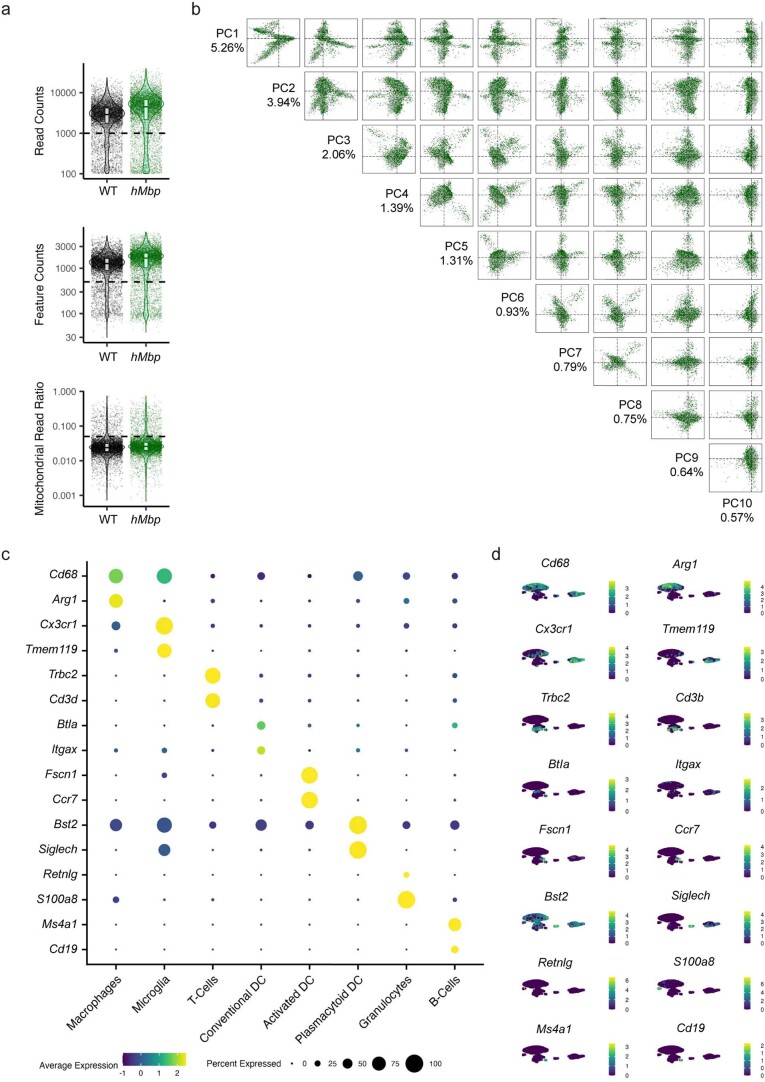
Quality control (QC) and cell type determination in single-cell RNA sequencing of immune cells in EAE. **a**, QC Violin plots showing UMI counts, feature counts, and the ratio of mitochondrial gene reads and chosen cut-offs. Single dots in the graphs represent single cells. N=1 biologically independent animal per group. Box bounds are according to the standard tukey format set as default values in ggplot2. Upper box bound: 75% percentile / centre mark: 50% percentile (median) / lower box bound: 25% percentile. Whiskers/Outliers/95% percentiles are not shown. **b**, Pairsplot of first 10 dimensions from a principle component (PC) analysis colored by genotype demonstrates no differential clustering between wildtype (black) and *hMbp* mice (green). **c**, Dot plot displaying the genes used for the identification of immune cell types. **d**, UMAP plots showing marker gene expression in the appropriate clusters.

**Extended Data Fig. 4:**
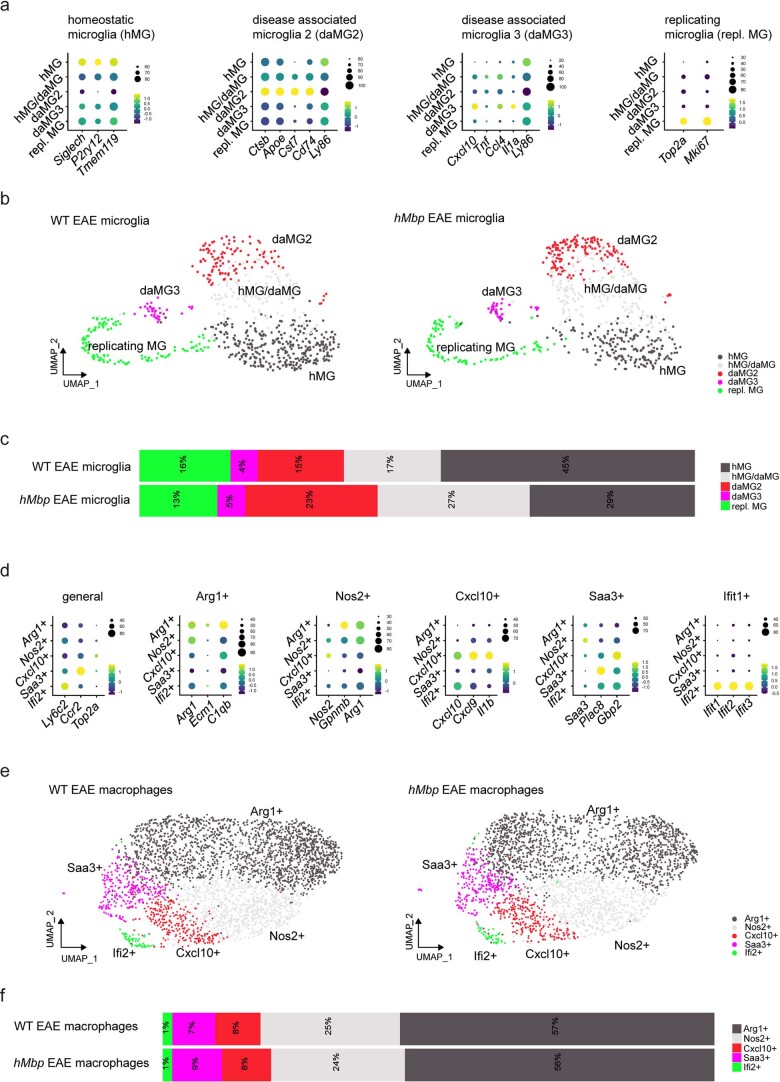
Subclustering of microglia and macrophage populations in EAE. **a-c**, Subclustering of the microglial cell populations revealed five subclusters in wildtype and *hMbp* mice at EAE disease peak (for an outline of the experiment see Fig. 4g), namely homeostatic microglia (hMG), disease-associated microglia (daMG2 and daMG3), a functional phenotype characterized by a gradient between hMG and daMG (hMG/daMG) and replicating microglia (repl. MG). **a**, Dotplots indicate relevant marker gene expression of the individual subpopulations as previously identified by Jordão *et al*.^39^. The colors indicate the scaled expression of marker genes while the dot size reflects the percent of cells expressing the marker gene. **b**, UMAPs visualizing the integrated subset of microglial for wildtype and *hMbp* mice independently, demonstrating a similar profile for both genotypes. **c**, Stacked bar plots representing the relative contribution of the five individual microglia subclusters to the total population, shown for wildtype and *hMbp* mice. **d-f**, Subclustering of the macrophage population identified five subclusters in wildtype and *hMbp* mice, namely *Arg1+*, *Nos2+*, *Cxcl10+*, *Saa3*+ and *Ifit1 +* clusters. **d**, Dotplots indicate relevant marker gene expression of the individual subpopulations as previously identified by Giladi *et al*.^40^. The colors indicate the scaled expression of marker genes and the dot size reflects the percentage of cells expressing the marker gene. **e**, UMAPs visualizing the integrated subset of macrophages for wildtype and *hMbp* mice, respectively, demonstrating similar profiles for both genotypes. **f**, Stacked bar plots representing the relative contribution of the five individual macrophage subclusters to the total population, shown for wildtype and *hMbp* mice.

To rule out underlying immunization differences, we compared the immunological response of *hMbp* EAE mice to respective wild-type EAE controls. We first confirmed that, in contrast to the reduced MBP expression, MOG protein levels (and hence EAE antigens) were unaltered in *hMbp* mutants (Supplementary Fig. 5b). We next performed fluorescent-activated cell sorting (FACS) analyses and immunohistochemistry to characterize immunological parameters in wild-type and *hMbp* mice upon EAE. Here, we found no major differences with respect to T cell and myeloid cell subtypes and numbers at disease peak (Supplementary Fig. 6a–h). To further corroborate these findings, we analyzed the immune cell activation profile of wild-type and *hMbp* mice on the individual cell level at disease peak by taking advantage of scRNA-seq (Fig. 6a and Extended Data Fig. 3a). Importantly, in a principal component analysis, none of the first ten principal components revealed a difference with regard to the genotype (Extended Data Fig. 3b). Likewise, unsupervised clustering (visualized using UMAP) of all immune cell populations demonstrates congruency between *hMbp* and wild-type mice (Fig. 6b). Major activation markers for microglia, macrophages and T lymphocytes as well as transcripts encoding key inflammatory cytokines were induced in EAE but did not show a genotype-dependent difference (Fig. 6c–f and Extended Data Fig. 3c,d). In line with this finding, a subcluster analysis for microglia (Extended Data Fig. 4a–c) and macrophages (Extended Data Fig. 4d–f) revealed no evidence of an attenuated immune response in *hMbp* mice compared to wild-type controls but demonstrated an overall similar cellular stoichiometry in microglia and macrophage subclusters in both genotypes (Extended Data Fig. 4).

**Fig. 7:**
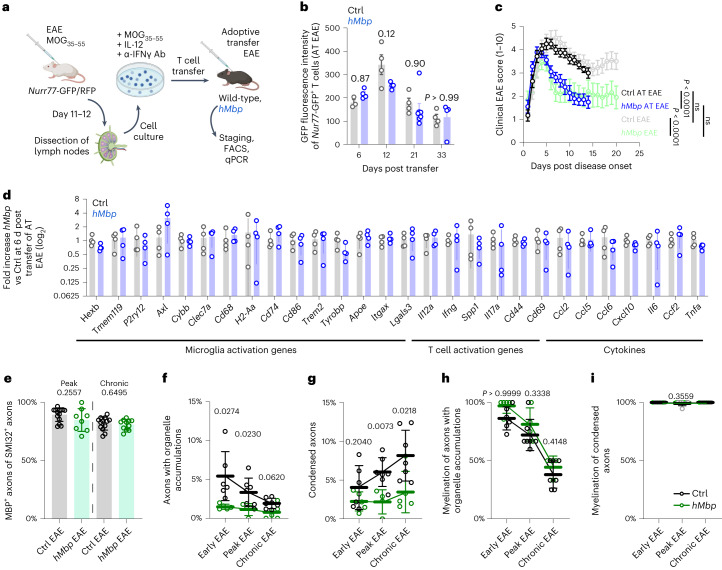
Axonal pathology is reduced in *hMbp* mice in EAE. **a**, Outline of the adoptive transfer EAE experiment. EAE was induced in *Nur77*-GFP/RFP mice, and after 11–12 d, the lymph nodes were dissected and the purified lymphocytes cultured with MOG and cytokines. Cultured T lymphocytes were then transferred to either wild-type (Ctrl) or *hMbp* mice to induce adoptive transfer EAE. Ab, antibody. **b**, Quantification of the mean fluorescence intensity of GFP of transferred CD4^+^ T cells as a measure for T cell activation indicated by *Nur77* expression by FACS reveals no difference in T cell activation between *hMbp* mice and controls. Two-way ANOVA with Sidak’s multiple comparisons test. AT, adoptive transfer. **c**, Clinical EAE scoring (1–10) after adoptive transfer shows an ameliorated disease course in *hMbp* mice compared to controls, reminiscent of the findings using classical EAE (Fig. 5c, light colors). Ctrl AT, *n* = 14; *hMbp* AT, *n* = 18. Pairwise two-way ANOVA. **d**, Quantification of marker mRNA expression by rt–qPCR for microglia and T cell activation and for relevant cytokines demonstrates no differences between Ctrl and *hMbp* mice. *n* = 4 per group, unpaired two-tailed Welch’s *t*-test. **e**, The percentage of myelinated SMI32^+^ axons is comparable between *hMbp* and Ctrl mice. For peak EAE, Ctrl, *n* = 14; *hMbp*, *n* = 8; for chronic EAE, Ctrl, *n* = 12; for *hMbp*, *n* = 11. Unpaired two-tailed Mann–Whitney test for each time point. **f**, The percentage of axonal organelle accumulations in electron micrographs of EAE lesions shows a reduction in *hMbp* mice compared to controls. **g**, *hMbp* mice demonstrate a lower percentage of axons with condensed axoplasm compared to controls at peak and chronic EAE. **h**, The myelination status of axonal organelle accumulations in *hMbp* and control mice reveals no difference between genotypes. **i**, In both *hMbp* and wild-type controls, condensed axons are an almost exclusive feature of myelinated axons. In (**f**–**i**), unpaired two-tailed Welch’s *t-*test was used for each time point. The key for **f**–**i** is given in **i**. For all graphs, circles represent biologically independent animals. Data are means ± s.d. except in **c**, in which data are means ± s.e.m.

Finally, to independently verify these results, we performed an adoptive transfer EAE experiment using T cells from reporter mice that carry a ubiquitous red fluorescent marker (RFP) in addition to an activation-dependent GFP biosensor (from the *Nur77* locus^30^) (Fig. 7a). Although RFP is ubiquitously expressed, T cell re-activation within the CNS tissue triggers a transient GFP expression in transferred T cells, which can be evaluated by flow cytometry (Fig. 7a,b). Importantly, we did not detect differences in T cell activation throughout the disease course between control and *hMbp* mice (Fig. 7b). Likewise, transcriptional analyses for major microglia and T lymphocyte activation markers as well as for respective inflammatory cytokines were similar between both genotypes (Fig. 7d). However, when following the clinical disease course in *hMbp* and control animals upon adoptive transfer, we were able to confirm a significantly ameliorated disease course in *hMbp* mutants (Fig. 7c). Indeed, the disease peak scores as well as the disease course were similar in both genotypes between the direct MOG_35−55_ EAE and the adoptive transfer EAE experiment (Fig. 7c and Supplementary Fig. 6i). These observations confirm that the improved clinical course and reduced axonal damage cannot be primarily explained by a difference in the immune response in *hMbp* mice and prompted us to further investigate the distribution of axon damage within EAE lesions of *hMbp* mutants. Here, despite the higher number of unmyelinated axons, axonal damage remained largely restricted to myelinated axons, as shown by co-immunostaining for SMI32 and MBP (Fig. 7e). Consistent with this finding, the percentages of axons with organelle accumulations and highly condensed axoplasm were both lower in *hMbp* mice than in controls, whereas the myelination status of axons with either pathology did not differ between genotypes and followed a similar time course (Fig. 7f–i). In summary, adoptive T cell transfer and in-depth immune cell profiling suggest that differences in inflammation are highly unlikely to account for the milder disease course and improved histopathology upon EAE in *hMbp* mice. Indeed, myelinated, not unmyelinated axons are primarily affected by irreversible damage in autoimmune lesions.

## Discussion

**Extended Data Fig. 5:**
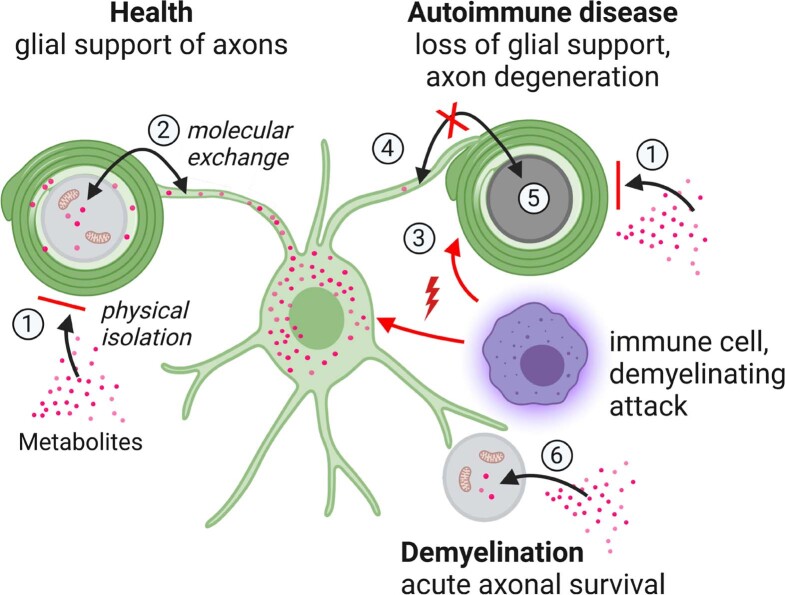
Working model on the role of myelin in inflammatory demyelinating disease. Myelinated axons are physically isolated from the environment by the myelin sheath (1) and therefore dependent on oligodendroglial support, for example metabolic exchange and detoxification (2). An autoimmune attack against myelinating oligodendrocytes by immune cells (3) perturbs glial support (4). Axons that are trapped in myelin insulation by dysfunctional oligodendrocytes are specifically vulnerable for irreversible degeneration (5). Rapid demyelination, in turn, acutely secures axonal survival (6).

Taken together, our data support a revised working model for inflammatory demyelinating lesions. We propose that the normally symbiotic relationship between the axon and the myelinating oligodendrocyte turns into a fatal one upon disease onset (Extended Data Fig. 5), and here show that persistent myelin insulation increases the risk of axon degeneration in an acute autoimmune inflammatory environment.

Consistent with previous studies^5,6,25,31,32^, we found that potentially reversible axonal organelle accumulations in not-yet demyelinated axons characterize early disease stages. Of note, organelle accumulations have been associated with axonal transport stasis in myelin mutants and shown to prevail at paranodal regions^33,34^. In line with this, we identified organelle accumulations developing locally at myelinated axon segments, while overall axon continuity (without signs of axonal constrictions) was preserved. Adjacent, already demyelinated segments of the same axon often appeared normal. Indeed, our data suggest that efficient demyelination can resolve axonal organelle accumulations—and hence determine the axon’s fate—by preventing the progression of axonal organelle accumulations to irreversible axonal degeneration. In contrast to axonal organelle accumulations, we demonstrate that axons with highly condensed cytoplasm are an almost unique feature of myelinated axons in autoimmune lesions. The condensed axoplasm reflects a disintegration of organelle membranes and cytoskeletal components, and longitudinal studies in models of Wallerian degeneration and other injury paradigms have defined this pathology as a sign of end-stage axonal degeneration^18–23^. A local disintegration of the axoplasmic components is thus likely to represent or eventually cause overall axonal degeneration. Notably, irreversible axonal damage remains restricted to myelinated axons in toxin-induced models with extensive demyelination and even in experimental models with a basal larger number of unmyelinated axons. Together with the higher prevalence of end-stage-damaged axons at the lesion border, this suggests that incomplete demyelination and, hence, axons with retained myelin rather than remyelinated axons are prone to irreversible degeneration.

We note that lesion development and inflammation are characterized by temporal dynamics, which we propose as pivotal in defining the role of myelin insulation for axonal function. Although myelin insulation by pre-existing, damaged oligodendrocytes turns fatal for axonal integrity in an acute inflammatory environment, upon inflammation resolution, the remyelination of surviving demyelinated axons by non-injured oligodendrocytes will remain essential for re-establishing axonal conduction properties and long-term axonal function.

Concordantly, although axonal myelination enables saltatory impulse propagation, myelin per se does not support axon integrity^16,17,33^. Hence, we provide a model in which immune-mediated damage to the myelinating oligodendrocyte results in defective axon–glia interactions and downstream axonal pathology (Extended Data Fig. 5). The concept of axonal pathology resulting from impaired axon–glia interactions is in agreement with findings in oligodendroglial mutants^10,15–17^ and transplantation experiments^9,25,33,34^, and is consistent with our understanding of oligodendroglial functions, such as axonal metabolic support^11–13,35,36^. Specifically, a role for oligodendrocytes in providing trophic support to the myelinated axon via non-compact myelinic channels and glial monocarboxylate transporters recently emerged^9,11–13,16,26,37^. Fully myelinated axons would hence be most vulnerable to autoimmune attacks that impact the capacity of the oligodendrocyte to metabolically support the myelinated axon. Consistent with this concept, diseased oligodendrocytes in EAE show a decreased expression of monocarboxylate transporter in scRNA-seq studies (Supplementary Fig. 4)^27,28^. In addition, we detected aberrant oligodendroglial cytoplasmic inner tongues associated with myelinated, irreversibly damaged axons (Supplementary Fig. 3g), potentially reflecting impaired trafficking of glial-associated metabolites to the glia–axon junction^11,16,25,26^. Untangling the molecular mechanisms by which immune-injured myelinating oligodendrocytes convey axonal damage may be key to identifying new axo-protective therapeutic targets in multiple sclerosis. Next to a potential loss of adequate trophic support, impairment of other glial functions such as detoxification^14^ may contribute to the specific vulnerability of myelinated axons in autoimmune demyelination^27,28^. In line with this premise, scRNA-seq studies of myelinating oligodendrocytes in EAE revealed a profound dysregulation of cellular processes implicated in cellular metabolism, cell stress and the glial immune response^27,28^ (Supplementary Fig. 4), which may help to uncover relevant oligodendroglial pathomechanisms in future studies. Interestingly, these perturbed oligodendroglial transcriptional signatures have also been identified in human multiple sclerosis^38^ and were found to predominantly map to the border of subcortical lesions, which is in support of our findings.

We emphasize that our model of myelin insulation as a risk factor for axonal integrity under inflammatory conditions is not mutually exclusive with recent studies on the role of reactive oxygen and nitrogen species and intra-axonal calcium levels in mediating axonal injury in EAE^5–7^, and we note that disease mechanisms in autoimmune demyelinating diseases involve a multitude of intertwining pathomechanistic cascades, which are likely to depend on temporal disease dynamics. In detail, axons isolated by dysfunctional oligodendrocytes are also likely to suffer from a reduced capacity to cope with additional injury-associated signals, such as reactive oxygen and nitrogen species, and therefore to show a higher baseline vulnerability compared to their denuded counterparts. Finally, although we suggest that rapid and efficient demyelination is protective in early autoimmune disease stages, chronic demyelination may impose independent additional risks to axons and impair axonal function, underlining the importance of promoting remyelination in multiple sclerosis lesions. Taken together, our study suggests that not demyelination per se, but oligodendroglial integrity and downstream axonal support may constitute crucial future therapeutic targets for acute autoimmune demyelinating diseases such as multiple sclerosis.

## Methods

### Human material

Analysis of human multiple sclerosis samples was performed on resin-embedded archival brain tissue from patients with multiple sclerosis obtained from the archives of the Institute of Neuropathology, University Medical Center Göttingen, Germany, and the Institute of Neuropathology, University Clinic Freiburg, Germany. Samples were anonymized and processed in a blinded manner. Epon-embedded tissue was cut into 50 nm sections and processed as described below (see Electron microscopy). Electron microscopy images underwent detailed neuropathological examination and *n* = 4 lesions (L1, L2, L3 and L4) from four individual patients were selected for further analyses based on the neuropathological criteria of inflammatory infiltrates (acute, active lesions) with either near-complete demyelination (L1), extensive demyelination (L2) or moderate demyelination (L3) and a lesion border (L4) with only minor signs of inflammation. Samples from L1 and L2 were each derived from a biopsy of the parietal lobe (white matter), and L3 and L4 were from a biopsy of the frontal lobe (white matter). The age at biopsy was between 18 and 31 for the four patients. The time of biopsy coincides with the initial diagnosis of multiple sclerosis for patients 1 and 2; patients 3 and 4 had relapsing–remitting multiple sclerosis disease at the time of biopsy. None of the patients had relevant comorbidities at the time of biopsy. Quantification was performed using FIJI 1.53c software^39^. All investigations were performed in compliance with relevant laws, institutional guidelines and the guidelines of the ethics committee of the University Medical Center Göttingen and the University Clinic Freiburg.

### Animal models

All mice were bred in a temperature-controlled room at 21 °C and 55% humidity with defined 12 h light and dark cycles and food and water ad libitum. Samples sizes are indicated for each individual experiment in the figure captions. C57BL/6J mice were used as control wild-type animals. *hMbp* mice are described elsewhere in detail and are characterized by >50% reduced expression of *Mbp*^19^. *Nur77*-GFP-positive mice are characterized elsewhere^30^. Mice between 10 and 14 weeks of age were used. For genotyping, DNA was isolated from tail biopsies and incubated in modified Gitschier buffer with Triton-X 100 and proteinase K for 2 h at 55 °C followed by heat inactivation of proteinase K for 10 min at 90 °C. Primer sequences are available upon request. All experiments were conducted according to either the Lower Saxony State regulations for animal experimentation in Germany as approved by the Niedersächsische Landesamt für Verbraucherschutz und Lebensmittelsicherheit (LAVES) and in compliance with the guidelines of the Max Planck Institute of Experimental Medicine, Göttingen, or approved by the Landesdirektion Sachsen and in compliance with the guidelines of the Paul Flechsig Institute, Leipzig.

### Classical EAE

EAE induction and staging were performed as described elsewhere^40^. Mice were kept in the room in which the procedure would take place for at least 2 weeks to allow acclimatization. On the day of immunization, mice were anesthetized with isoflurane, and an emulsion of Complete Freund’s Adjuvant (CFA), made from *Mycobacterium tuberculosis* (BD Bioscience, 231141) with a total of 200 µg MOG_35–55_ (AnaSpec, AS-60130-1) per mouse, was injected subcutaneously into all four flanks of each mouse. A total of 400 ng of pertussis toxin (Sigma, P7208) was administered intraperitoneally after immunization and 48 h later. Mice were weighed and scored every day at the same time according to EAE scoring system from score 0 to 10 as previously published^40^. Mice that had disease onset after day 20 were excluded. Mice that exceeded a score of 4 were put on special bedding for easier movement and water gel and food were put inside the cage for easier accessibility. Both female and male mice were used, and both sexes recapitulated the significant differences in the EAE course between wild-type and *hMbp* mutants.

### Adoptive transfer EAE

For EAE induction through adoptive transfer of pathogenic T cells, donor *Nur77*-GFP/RFP (*Nur77*-GFP and ubiquitous RFP) mice were immunized with 75 µg of MOG_35–55_ per mouse in CFA. A total of 200 ng of pertussis toxin was administered intraperitoneally on day 0 and day 2 post immunization. Draining lymph nodes were collected on day 12 post immunization. A single-cell suspension was prepared, and the cells were cultivated for 3 d in the presence of 25 µg ml^–1^ MOG_35–55_, 25 ng ml^–1^ recombinant mouse IL-12 (R&D, 419-ML) and 20 µg ml^–1^ α-IFNу-Ab (BioXcell, clone XMG1.2, BE0055). To induce EAE, 3 × 10^6^ cells were injected intraperitoneally into recipient *hMbp* or wild-type animals with the same genetic background. Clinical signs and body weights were examined daily starting on day 4 post T cell transfer. On days 6, 12, 21 and 33 post transfer, 3–6 animals were killed and T cell activation was quantified using flow cytometry. Therefore, from *n* = 14 control animals undergoing adoptive transfer EAE and clinical scoring, two animals were killed 7 days post (disease) onset (dpo), two animals were killed 8 dpo and one animal was killed 13 dpo. Hence, nine animals were followed until 14 dpo. For *hMbp* mutants, a total number of *n* = 18 animals underwent EAE adoptive transfer, with three animals killed 7 dpo and one 8 dpo. Hence, 14 animals were followed until 14 dpo. As in classic EAE, mixed female and male mice were used, with both sexes recapitulating the significantly ameliorated disease course of *hMbp* mutants compared to controls.

### CPZ-induced demyelination

CPZ treatment was performed as described previously^41^. CPZ (0.2% w/w; Sigma, C9012) was fed as powder chow, and mice were killed at four time points: day 0 (control without CPZ), 3 weeks of CPZ treatment (demyelination onset), 5 weeks of CPZ treatment (progressed demyelination) and 12 weeks of CPZ treatment (full demyelination). Food intake and weight were monitored. Animals were perfused with PBS, followed by 2.5% glutaraldehyde and 4% paraformaldehyde (PFA) in 0.1 M phosphate buffer for electron microscopy. Fixed brains were cut using a vibratome (Leica VT1200, 300 mm) and the corpus callosum with adjacent tissue was punched with a 2 mm punching tool and processed as described for electron microscopy. For electron microscopy analysis and axonal counting, axons smaller than 400 nm in diameter were excluded from quantification. Only male mice were used.

### LPC-induced demyelination

Stereotactic injection of LPC (Sigma, L4129) was performed as described previously^42,43^. Mice were anesthetized intraperitoneally with MMF (0.5 mg medetomidin per kg, 5.0 mg midazolam per kg and 0.05 mg fentanyl per kg). The head fur was trimmed and the eyes covered with Bepanthene cream (Bayer). A small incision was made to expose the skull and a hole was drilled ±1.0 mm (*X*) and −0.1 mm (*Y*) from bregma. A capillary filled with 1% LPC and 0.03% Monastral Blue (Sigma, 274011) for lesion visualization was inserted into the corpus callosum −1.4 mm (*Z*) from bregma, using a micromanipulator (Nanoliter 2000, World Precision Instruments); 1 µl was injected with a flow rate of 100 nl min^–1^. After the injection, the head skin was sutured and 0.05 mg kg^–1^ of buprenorphine was administered. Anesthesia was antagonized with AFN (2.5 mg kg^–1^ atipamezol, 1.2 mg kg^–1^ naloxone, 0.5 mg kg^–1^ flumazenil) and mice were closely monitored. Only male mice were used.

### FACS standard EAE

Mice were perfused at EAE peak (4 dpo) with PBS and heparin after blood was taken from the right ventricle into an EDTA pre-filled tube on ice. The spinal cord was dissected and dissociated with a scalpel on a drop of medium containing Hanks’ balance salt solution, glucose and HEPES. The cell solution was filtered through a 40 µm strainer, centrifuged, resuspended in 75% Percoll (GE Healthcare) and layered under a 25% Percoll solution topped with PBS. After centrifugation for 30 min with slow breaking, distinct gradients became visible and myelin debris was carefully removed. The remaining cell phase was taken up, washed by centrifugation with PBS and stained with viability dye (eFluor 506 Fixable Viability Dye, Thermo Fisher) for 30 min on ice. In parallel, erythrocyte lysis using erythrocyte lysis buffer (BD Pharma) was performed for blood samples. For both sample types, cells were blocked with Fc block (CD16/32, Invitrogen, 14-0161-82) for 20 min on ice. After washing, antibodies were incubated for 20 min on ice. For lymphocytes, CD45^+^ CD11b^−^ cells were gated and differentiated using CD4 and CD8. CD45^+^ CD11b^+^ myeloid cells were further differentiated into neutrophils (Ly6C^hi^, CD115^−^) and two populations of monocytes (Ly6C^hi^ or Ly6C^lo^, CD115^+^). The following antibodies were used: CD11b-BV421 (1:200, BioLegend, 101236), Ly6C-Alexa488 (1:200, BioLegend, 128022), CD4-PE (1:200, BioLegend, 100512), CD8b-Alexa647 (1:200, BioLegend, 126611), CD45-APC-e780 (1:200, Invitrogen, 47-0451-82) and CD115-PE-Cy7 (1:100, Invitrogen, 25-1152-82). FACS analysis was performed using a FACS Aria III system (BD Biosciences) and data were analyzed using FlowJo software (Tree Star).

### FACS adoptive transfer EAE

For analysis of adoptive transfer EAE spinal cord tissue, mice were perfused with PBS, and the spinal cord was digested with Liberase (Roche) and DNase I (Sigma) for 15 min at 37 °C in a water bath. Subsequently, a single-cell suspension was prepared and myelin was removed by Percoll gradient (40%) centrifugation. After centrifugation, the cell pellet was resuspended in FACS buffer and filtered through a 40 µM filter. The cells were blocked using an Fc blocking antibody (BioLegend Fc-block clone, 2.4G2) and lymphocytes were stained with CD3 and CD4. All FACS antibodies were purchased from BioLegend: CD4-PE-Cy5 (1:200, 100409, clone GK1.5) and CD3e APC (1:200, 100311, clone 145-2C11). CD4^+^ T cells (CD3^+^CD4^+^) were divided into transferred (RFP^+^) and endogenous (RFP^−^) T cells. T cell activation was measured by the level of GFP expression (geometric mean fluorescent intensity) of transferred T cells. The data were analyzed using FlowJo software (Tree Star).

### Electron microscopy

#### 2D electron microscopy

Mice were perfused with PBS, and two lumbar spinal cord segments were dissected and fixed in 0.1 M phosphate buffer containing 2.5% glutaraldehyde and 4% PFA for at least 1 week at 4 °C. Fixed samples were post-fixed using 1% osmium tetroxide for 1 h and dehydrated using increasing concentrations of ethanol. For contrast enhancement, samples were incubated with 1% uranyl acetate for 1 h at room temperature (20 °C). Samples were infiltrated in increasing amounts of Agar 100 epoxy resin (Agar Scientific) and polymerized in Agar 100 resin for 48 h at 60 °C. Then, 500 nm semi-thin sections were stained with a methylene blue–azure II solution for 1 min on a 60 °C heating plate. Ultrathin sections (50 nm) were taken up with formvar-coated grids and contrasted with Uranlyess (Delta Microscopies) and lead citrate. Images were taken with a SIGMA electron microscope (Zeiss) equipped with a scanning transmission electron microscope detector and ATLAS software. For each lesion, at least 15 images of ×4,000 magnification were analyzed using FIJI^39^ software. Specific parameters were defined for axons with organelle accumulations (characterized by a higher number and different sizes of mitochondria and more than one abnormal vesicular structure) and dense axons (characterized by noticeably darker cytoplasm and disintegration of axoplasmic components compared to neighboring axons). For analysis of the non-compact adaxonal myelin (the inner tongue), at least 40 normally myelinated axons were analyzed in each control spinal cord. In EAE lesions, at least 40 normally myelinated axons and 10–20 condensed axons were analyzed per animal. The area of the inner tongue was measured and expressed relative to the area of the respective axon.

#### Sample processing and reconstruction for 3D electron microscopy

To prepare samples for 3D electron microscopy, mice were flushed at 4 dpo with 0.1 M phosphate buffer, and the lumbosacral spinal cord was dissected, sectioned in 2–3-mm-thick pieces and fixed in 0.1 M phosphate buffer containing 2.5% glutaraldehyde and 4% PFA for at least 1 week at 4 °C. Subsequently, samples were post-fixed and contrast-enhanced using reduced osmium (2% OsO_4_ and 0.5% Kaliumhexacyanoferrat) for 3 h at 4 °C followed by incubation for 1 h at room temperature in 1% thiocarbohydracide and 1.5 h at room temperature in 2% OsO4 followed by overnight incubation in 2% uranyl acetate at 4 °C. The next day, samples were dehydrated by increasing steps of acetone. Finally, samples were infiltrated with increasing steps of Durcupan in acetone and polymerized for 48 h at 60 °C in pure Durcupan (Sigma). Semi-thin sections (500 nm) allowed identification of lesions and definition of regions of interest to be further processed in 3D. Therefore, cubes of approximately 1 mm in *X* and *Y* dimensions and 500 nm in depth were trimmed and mounted on scanning electron microscopy stubs (Science Services). After scanning the whole block-face for quality control and to define a region of interest, serial block-face scanning electron microscopy was performed with a Zeiss Merlin VP compact scanning electron microscope (Carl Zeiss Microscopy) equipped with a Gatan 3View2XP System (Gatan). Images were acquired at 3 kV and 30 Pa in variable pressure mode, with 1 µs dwell time and 15 nm pixel size. The section thickness was 80 nm and the block-face was imaged every third section, resulting in *Z*-steps of 240 nm between acquired images.

The image sequence was subsequently converted to an 8-bit Tiff, followed by local contrast enhancement (contrast limited adaptive histogram equalization (CLAHE)), smoothing by Gaussian blur with Sigma 1.5 and brightness and contrast adjustment using FIJI^39^. For alignment, the drift correction algorithm correlating with the previous slice was performed using Microscopy Image Browser^44^. For subsequent segmentation, the brush and black and white thresholding algorithms were combined in Microscopy Image Browser to separately segment the myelin from the axon and organelles. The results of the segmentations were saved as a 2D sequence and imported into IMARIS v.9.9 (Oxford Instruments), and each segmentation result was interpreted as a surface that allowed for visualization of the different structures.

#### Immunohistochemistry and imaging

For immunohistochemistry, mice were perfused with PBS, and two lumbar spinal cord segments were dissected and post-fixed in 4% PFA in PBS overnight at 4 °C. Samples were embedded in paraffin and cut into 5 µm sections. Samples were de-waxed in xylol and ethanol, antigen retrieval was performed using boiling citrate buffer (pH 6) and samples were blocked with the serum of the secondary antibody host species. Primary antibodies were applied overnight at 4 °C and included SMI31 (1:1,000, BioLegend, 801601), IBA1 (1:400, Wako, 019-19741), SMI32 (1:400, BioLegend, 801701), MBP (1:2,500, Invitrogen, PA1-10008), APP (1:1,000, Merck, MAB348), NEUN (1:100, Merck Millipore, MAB377) and CD3 (1:100, Abcam, ab5690). For fluorescent stainings, secondary antibodies were applied for 1 h (all antibodies were purchased from Dianova and used at 1:1,000) and included Cy2 goat anti-mouse (115-225-071), Cy2 goat anti-rat (112-225-143), Cy2 goat anti-rabbit (111-225-144), Cy3 goat anti-mouse (115-165-071), Cy3 goat anti-rat (112-165-003), Cy3 goat anti-rabbit (111-165-144) and Cy3 goat anti-chicken (303-165-003). The samples were then counterstained with DAPI (Invitrogen, D1306) and embedded in AquaPolymount (Polysciences, 18606). For chromogenic stainings, primary antibodies were detected using biotin-coupled secondary antibodies (all antibodies were purchased from Southern Biotech: goat anti-mouse (1012-08), goat anti-rat (3052-08)) for 1 h at room temperature, and labeled structures were visualized using the ABC Vectastain kit (Vector laboratories, PK-6100). Finally, samples were counterstained with hemalum and embedded in Eukitt (O-Kindler). Overview pictures for quantification of larger structures (for example, cells) were taken with the Zeiss AxioScan Z1. High-resolution images for more detailed analyses (for example, SMI32 MBP correlation) were taken with a Zeiss LSM 880 Airyscan confocal microscope. In both cases, images were acquired with Zen imaging software (Zeiss) and analyzed using FIJI^39^. To visualize 3D reconstructions of damaged axons, *Z*-stacks were acquired with a ×63/1.4NA objective with a step size of 0.17 µm. Subsequently, the *Z*-stacks were processed using IMARIS software and separate surfaces for the SMI32^+^ and the MBP^+^ signal were created.

#### Western blot

To quantify protein amounts, 2-month-old mice were intracardially perfused with 1× PBS using a peristaltic pump (World Precision Instruments) for 3 min. Then, the spinal cord was removed and dissected to isolate the lumbosacral ventrolateral white matter, which was subsequently flash-frozen on dry ice. The tissue was lysed with fresh SDS lysis buffer with Mini Complete protease inhibitor and PhoStop phosphatase inhibitor (Roche) and homogenized with Precellys 24. A final protein concentration of 1 mg ml^−1^ was prepared in Laemmli buffer and 10 µg of protein was loaded per slot. The proteins were separated on a 12% Bis-Tris mini protein gel and run for 10 min at 80 V and 45 min at 200 V. Subsequently, proteins were transferred onto a PVDF membrane and detected using antibodies (MOG: Abcam, ab109746, rabbit, 1:2,000; secondary HRP-anti-rabbit, Cell Signaling, 7074 S, 1:15,000; MBP: Sigma, Atlas, AMAB91064, mouse, 1:5,000; secondary HRP-anti-mouse, Cell Signaling, 7076, 1:5,000; ACTB: Sigma, A5441, mouse, 1:2,500; secondary HRP-anti-mouse, Cell Signaling, 7076, 1:5,000). The antibodies were incubated separately and subsequently stripped using glycin, SDS and Tween for 10 min before proceeding with the next antibody. Detection was performed using ECL.

#### Quantitative PCR analysis

Expression of mRNA was quantified in whole lumbar spinal cord cell suspensions derived from adoptive transfer EAE at 6 dpo. Cell suspensions were transferred into 1.5 ml Eppendorf tubes and resuspended in QIAzol Lysis Reagent (Qiagen Sciences) and frozen at −80 °C. For RNA isolation, samples were incubated with 60 µl of chloroform for 20 min and subsequently incubated with 150 µl of isopropanol (Roth) and 1 µl of glycogen (Roche) for 10 min. After centrifugation, the supernatant was removed and the pellet was washed twice with 75% ethanol and then dried. For conversion to cDNA, the RevertAid First Strand cDNA Synthesis Kit (Thermo Scientific) was used, following the manufacturer’s protocol. Quantitative PCR was performed using the BRYT-Green system (A6002, Promega) on a qPCR qTOWER84 (Jena Analytics). The concentration of primers was kept at 300 nM and all samples were run in quadruplicate. *Pgk1*, *Sdha* and *Rps26* were used as housekeeping primers. Primer sequences are available upon request.

#### Sample and library preparation for scRNA-seq

To prepare immune cells for scRNA-seq, two wild-type mice (one male, one female) and two *hMbp* mice (one male, one female) were perfused at 4 dpo (EAE disease peak) with Hanks’ balance salt solution without Ca^2+^, Mg^2+^ or Phenol Red (Gibco, Thermo Fisher Scientific, 14175-145) containing 5 µg ml^–1^ actinomycin D and 10 µM triptolide for 3 min. Then, the lumbosacral spinal cord was obtained and further dissected to isolate the ventrolateral white matter using fine knives under microscopic evaluation. The dissected lumbosacral ventrolateral white matter was dissociated to a single-cell suspension using a shortened protocol of the adult brain dissociation kit (ABDK, Miltenyi Biotech), in which three incubations of 5 min at 37 °C were interspaced by manual tissue dissociation using fire-polished glass pipettes of three different sizes. During tissue dissociation, 5 µg ml^–1^ actinomycin D, 27.1 μg ml^–1^ anisomycin and 10 µM triptolide were added to the enzymatic solution to inhibit transcription and translation as described previously^45,46^. From this point on, cells were kept on ice. The single-cell suspension was passed through a 70 µm cell strainer and debris were removed according to the ABDK instructions. Finally, cells were incubated for 15 min with magnetically labeled CD45 antibodies and then isolated using an MS column (miltenyi biotech). scRNA-seq on MACS-purified immune cells was performed using the 10× Genomics 3ʹ Single-Cell v.3.1 kit. The cell count in the single-cell suspension was quantified by manual hemocytometer cell counts, and the suspension was diluted to a cell stock concentration of 1,000 cells per µl. The appropriate volume of cell stock for a targeted cell recovery of 10,000 cells was mixed with nuclease-free H_2_O and the Master Mix according to the Chromium Single-Cell 3′ Reagent Kits v.3.1 (dual index) user guide. The cell suspensions were loaded onto Chromium Next GEM Chip G v.3 and run using a Chromium controller. Barcoded single-cell libraries were generated from droplets following the manufacturer’s specifications. The quality of the libraries was assessed using the Agilent 5200 Fragment Analyzer running the High Sensitivity DNA quantification kit.

#### Next-generation sequencing and single-cell data analysis

Single-cell libraries were prepared for sequencing using Illumina NextSeq550 and Novaseq 6000 according to the manufacturer’s guidelines and sequenced with 150 bp paired-end reads. Sequencing was performed by the Core Unit DNA-Technologien (Medical Faculty, Leipzig University) and the Institute for Human Genetics (University Hospital Leipzig). Raw Illumina BCL files were demultiplexed using the mkfastq command in bcl2fastq v.2.20. Gene expression matrices were generated with the count command in Cell Ranger v.3.1.0 using the default mm10 mouse genome bundled with Cell Ranger. scRNA-seq data were analyzed using R v.4.1.2 (https://www.r-project.org), primarily with the help of the package Seurat v.4.1.1 (ref. ^47^). Additional plotting was performed using ggplot2 v.3.3.6, scCustomize v.0.7.0 (https://zenodo.org/record/5834562#.YzhmeXZBzBQ) and PCAtools v.2.6.0 (ref. ^48^).

For quality control, cells were filtered to contain more than 1,000 unique molecular identifiers, 500 genes and less than 5% mitochondrial gene reads. Data were log-normalized and the 2,000 most highly variable genes were selected. Data were scaled and centered using the Seurat function Sacle Data, followed by linear dimensionality reduction using principal component analysis (PCA). Based on the ElbowPlot, the first 30 dimensions were selected for downstream analysis. Clustering was performed using the shared nearest neighbor modularity optimization approach^49^ implemented in Seurat with a resolution parameter of 0.8. Nonlinear dimensionality reduction was performed using UMAP^50^ with hyperparameters n.neighbors = 30 and min.dist = 0.3. At this point, iterative quality control was performed by calculating marker genes for every cluster using the Wilcoxon rank sum test with Bonferroni correction as implemented in Seurat. Contaminating cell populations consisting of oligodendrocytes (*Ptgds*^+^) and endothelial cells (*Cldn5*^+^) were excluded. Doublets were removed manually, on the basis of dual cell-type marker gene expression, and automatically, using scDblFinder v.1.8.0 and scran v.1.22.1. Cell cycle scores were calculated using a previously established method^51^ as described in the teaching materials at the Harvard Chan Bioinformatics Core (https://hbctraining.github.io/scRNA-seq_online/lessons/cell_cycle_scoring.html). The remaining cells were reprocessed as before, but regressing out the cell cycle phase during data scaling. Clusters were annotated based on plotting of canonical marker gene expression and a literature search^39,40,52–58^ (https://www.cellsignal.com/pathways/immune-cell-markers-mouse) of marker genes that were calculated using the Wilcoxon rank sum test as implemented in Seurat. Clusters were joined until each cell type was represented by one cluster.

Microglia and macrophage populations were selected individually and integrated by genotype using canonical correlation analysis as implemented in Seurat. Cell-type-specific datasets were rescaled, PCA was performed and the first 15 principal components were used for dimensional reduction with UMAP using the parameters stated above. Subclusters were identified using a resolution parameter of 0.5, and contaminating cell types were removed based on marker gene expression. The process was repeated, and subtypes were annotated based on the expression of marker gene sets according to previous studies^59,60^. The expression of canonical genes for homeostasis and activation of microglia and macrophages was plotted for their respective cell cluster using ggplot2. The expression of cytokines was plotted for all cells jointly. A pairsplot of the first ten dimensions of PCA, calculated using the top 2,000 variable genes as determined by FindVariableGenes using the vst method, was plotted using PCAtools v.2.6.0. Subpopulations of macrophages and microglia were highlighted by plotting the UMAP for both genotypes individually along with the expression of marker genes as reported in the literature. The relative proportion of cell subpopulations in each sample was visualized as a stacked bar plot using ggplot2.

#### Analysis of published data

For the re-analysis of published data, we recruited two scRNA-seq datasets^27,28^. In both, GFP^+^-sorted cells from control mice (injected with CFA) and mice at peak EAE (injected with MOG_35–55_ in CFA, EAE score = 3) were analyzed. Due to differences in sequencing technology and GFP reporter, the datasets were processed independently. The first dataset (GSE113973) was generated in 2018 (ref. ^27^). In brief, the authors sequenced FACS-sorted GFP^+^ cells from Pdgfra-H2B-GFP or Pdgfra-Cre::LoxP-GFP animals using the plate-based SmartSeq2 protocol. The processed count data were downloaded from GEO and annotations from the UCSC Cell Browser (https://cells.ucsc.edu/?ds=oligo-lineage-ms). The data were filtered to contain only cells annotated as mature oligodendrocytes based on annotation by the authors. For further quality control, data were rescaled, PCA was computed using the 2,000 most variable features followed by nonlinear dimensionality reduction using UMAP with first the ten principal components as input and standard parameters (Seurat v.4.1) before plotting canonical marker genes. One outlying plate was detected from both the UMAP space and the PCA of pseudo-bulk profiles, and was excluded from further analysis. The scRNA-seq data processing pipeline was repeated after removing the outlier sample. Differences in gene expression were determined by generating pseudo-bulks. Gene expression of all cells from individual samples was aggregated and the resulting count matrix was used as an input to DESeq2. Genes with less than ten reads were excluded from further analysis. The second dataset (GSE193238) was published in 2022 (ref. ^28^). In brief, the authors analyzed FACS-sorted GFP^+^ cells from Sox10-Cre::LoxP-GFP animals using the Multiome kit (10× Genomics). The processed scRNA-seq data were downloaded from GEO and filtered to contain only cells annotated as mature oligodendrocytes based on annotation by the authors. For further quality control, data were rescaled, and PCA was computed using the 2,000 most variable features followed by nonlinear dimensionality reduction using UMAP with the first 15 principal components as input and standard parameters (Seurat v.4.1) before plotting canonical marker genes. Differences in gene expression between control and EAE cells were calculated using the Wilcoxon rank sum test with Bonferroni correction for genes expressed in at least 10% of the cells in each group that differ in their log_2_-fold change by at least 0.1. For both datasets, DEG lists were filtered for adjusted *P* value (Bonferroni correction; *P* < 0.05) and split based on positive or negative log_2_-fold change (Supplementary Data 1). Individual gene lists were used for overrepresentation analysis using GProfiler2 (v.0.2.1) for the Gene Ontology:Biological Process (GO:BP) database. Significantly overrepresented GO terms were analyzed and visualized for their similarities using the R package simplifyEnrichment (v.1.8.0). Using the simplifyGOFromMultipleLists function, adjusted *P* values of overrepresented terms from each dataset are presented in a heatmap on the left panel, and correlations between the terms are presented in the middle panel. Based on their correlation, GO terms are then grouped into clusters using binary cut (default cutoff = 0.85), and keywords from each cluster are shown in the right panel. For a cleaner visualization, broad and general terms including ‘process’, ’biological’, ‘responses’ and ‘regulation’ were silenced from the keyword representation.

#### Statistical analysis and reproducibility

For power analyses, the software G*Power v.3.1.7. was used and performed before conducting in vivo experiments (a priori) to choose sample size. Adequate power (1 – beta error) was defined as ≥80% and the alpha error as 5%. Data are expressed as biologically independent samples with mean ± s.d. unless indicated otherwise. For animal studies, organisms were allocated randomly to the experimental groups, only considering the determined genotypes. EAE induction was performed in an alternating fashion between the cages of different (blinded) genotypes. For human studies, no grouping, and hence no randomization, was necessary. All samples were processed in a single-blinded manner. All data were processed and statistically analyzed using Microsoft Excel and GraphPad Prism v.7.05 unless indicated otherwise. All values obtained were included unless stated otherwise in the relevant Methods sections. The respective statistical tests that were used are indicated in the figure legends. In brief, normal distribution was tested using the D’Agostino-Pearson omnibus normality test or Shapiro–Wilk test. To compare two groups, the unpaired two-tailed Welch *t*-test (normal distribution) or Mann–Whitney test (no normal distribution) was used. To compare more than two groups, a one-way ANOVA with the Tukey’s multiple comparisons test (normal distribution) or the Kruskal–Wallis test with Dunn’s multiple comparisons test (not normal distribution) were used, and to compare two or more groups for more than one time point (longitudinal analysis), a two-way ANOVA with the appropriate post-hoc test was used. Equal variance was not formally tested and was assumed to be different between groups. Statistical differences were considered to be significant when *P* < 0.05 and are indicated as exact numbers in the graphs. Displayed micrographs in Figs. 1e, 2b–d, 3a, 4a, 5b,d,e, Extended Data Fig. 2a–d and Supplementary Figs. 2a–f, 3a,c–e,g and 6g,h are representative of at least three biologically independent samples. Figs. 1a, 2e,f, Extended Data Fig. 1a–c and Supplementary Fig. 1b are representative of one biologically independent sample. Schematic experimental diagrams were created using BioRender ((https://www.biorender.com).

### Reporting summary

Further information on research design is available in the Nature Portfolio Reporting Summary linked to this article.

## Online content

Any methods, additional references, Nature Portfolio reporting summaries, source data, extended data, supplementary information, acknowledgements, peer review information, details of author contributions and competing interests and statements of data and code availability are available at 10.1038/s41593-023-01366-9.

## Supplementary information

Supplementary Information Supplementary Figs. 1–6, Legends for Supplementary Videos 1–4, Supplementary Table 1, Legend for Supplementary Data 1.

Reporting Summary

Supplementary Video 1 3D segmentation of an axon with organelle accumulation.

Supplementary Video 2 3D model of an axon with organelle accumulation.

Supplementary Video 3 3D segmentation of an axon with condensed cytoplasm.

Supplementary Video 4 3D model of an axon with condensed cytoplasm.

Supplementary Data 1 Analysis for overrepresentation of Gene Ontology:Biological Process (GO:BP) annotations in published datasets of the oligodendrocyte response to EAE.

Supplementary Data 2 Uncropped Western blots for Supplementary Fig. 5b.

## Data Availability

All data relevant to the present manuscript are available from the corresponding authors on reasonable request. Sequencing data for all mouse samples generated for this study have been deposited in the NCBI GEO database (GSE222063). Seurat objects and Cell Ranger output files are available in the GEO supplement, and raw fastq files can be accessed from the Sequence Read Archive (links are provided in the GEO records). The expression data that we reanalyzed were also accessed from GEO (GSE113973 and GSE193238).

## Code availability

The code to reproduce figures can be found at https://github.com/nikofleischer/myelin-axonal-damage.
