# Addendum: Myelin insulation as a risk factor for axonal degeneration in autoimmune demyelinating disease

**DOI:** 10.1038/s41593-025-01927-0

**Published:** 2025-03-11

**Authors:** Erik Schäffner, Mar Bosch-Queralt, Julia M. Edgar, Maria Lehning, Judith Strauß, Niko Fleischer, Theresa Kungl, Peter Wieghofer, Stefan A. Berghoff, Tilo Reinert, Martin Krueger, Markus Morawski, Wiebke Möbius, Alonso Barrantes-Freer, Jens Stieler, Ting Sun, Gesine Saher, Markus H. Schwab, Christoph Wrede, Maximilian Frosch, Marco Prinz, Daniel S. Reich, Alexander Flügel, Christine Stadelmann, Robert Fledrich, Klaus-Armin Nave, Ruth M. Stassart

**Affiliations:** 1 Department of Neurogenetics, Max Planck Institute for Multidisciplinary Sciences, Göttingen, Germany; 2 Paul Flechsig Institute of Neuropathology, University Clinic Leipzig, Leipzig, Germany; 3 Institute of Infection, Immunity and Inflammation, College of Medical Veterinary and Life Sciences, University of Glasgow, Glasgow, UK; 4 Institute of Neuroimmunology and Multiple Sclerosis Research, University Medical Center Göttingen, Göttingen, Germany; 5 Institute of Anatomy, Leipzig University, Leipzig, Germany; 6 Cellular Neuroanatomy, Institute of Theoretical Medicine, Medical Faculty, University of Augsburg, Augsburg, Germany; 7 German Center for Neurodegenerative Diseases (DZNE), Munich, Germany; 8 Department of Neurophysics, Max Planck Institute for Human Cognitive and Brain Sciences, Leipzig, Germany; 9 Paul Flechsig Institute of Brain Research, Leipzig University, Leipzig, Germany; 10 Institute of Functional and Applied Anatomy, Research Core Unit Electron Microscopy, Hannover Medical School, Hannover, Germany; 11 Institute of Neuropathology, Medical Faculty, University of Freiburg, Freiburg, Germany; 12 Centre for NeuroModulation (NeuroModBasics), University of Freiburg, Freiburg, Germany; 13 Signalling Research Centres BIOSS and CIBSS, University of Freiburg, Freiburg, Germany; 14 Translational Neuroradiology Section, National Institute of Neurological Disorders and Stroke, National Institutes of Health, Bethesda, MD USA; 15 Institute of Neuropathology, University Medical Center Göttingen, Göttingen, Germany

Addendum to: *Nature Neuroscience* 10.1038/s41593-023-01366-9, published online 29 June 2023.

It has come to our attention that readers may misinterpret our study as questioning the axon-protective nature of myelin in general or as a caution against remyelination-promoting therapies for diseases such as multiple sclerosis. We want to clarify the data reported in our study do not question the protective nature of myelin in physiological conditions or the benefits of long-term remyelination.

In our study, we investigated the relationship between myelination and axonal pathology in human multiple sclerosis and its mouse models. We observed that persistent ensheathment of axons with myelin in an autoimmune environment constitutes a risk factor for irreversible axonal degeneration. These findings led us to propose a model in which the disruption of oligodendroglial health and their axonal support acts as an upstream effector of axonal damage. While our findings introduce a previously overlooked element of multiple sclerosis pathology, we explicitly noted that chronic demyelination may independently pose a long-term risk to axons. Accordingly, our statement that “rapid and efficient demyelination in early autoimmune disease may reduce the risk to axons of undergoing irreversible damage” does not undermine the axon-protective role of myelin under physiological conditions. Rather, we emphasized that only myelin insulation by dysfunctional oligodendrocytes, when retained, is detrimental for axons, whereas remyelination with intact myelin by healthy oligodendrocytes is crucial for restoring axonal conduction and ensuring axonal function. Our study highlights the need to identify novel therapeutic targets that address both myelin restoration and oligodendroglial health to preserve axonal survival and long-term integrity in multiple sclerosis and related disorders.

