# Supplementary material for: Myelin insulation as a risk factor for axonal degeneration in autoimmune demyelinating disease
Source: Nat Neurosci. 2023 Jun 29;26(7):1218–28. doi: 10.1038/s41593-023-01366-9 (PMC10322724; doi:10.1038/s41593-023-01366-9)
Supplement: Supplementary file 1 — Supplementary Figs. 1–6, Legends for Supplementary Videos 1–4, Supplementary Table 1, Legend for Supplementary Data 1. [file 41593_2023_1366_MOESM1_ESM.pdf]

nature neuroscience

Article https://doi.org/10.1038/s41593-023-01366-9

Myelin insulation as a risk factor for axonal degeneration in autoimmune demyelinating disease

In the format provided by the authors and unedited

Supplementary Information File for Schäffner&Bosch-Queralt *et al*.

Supplementary Figures

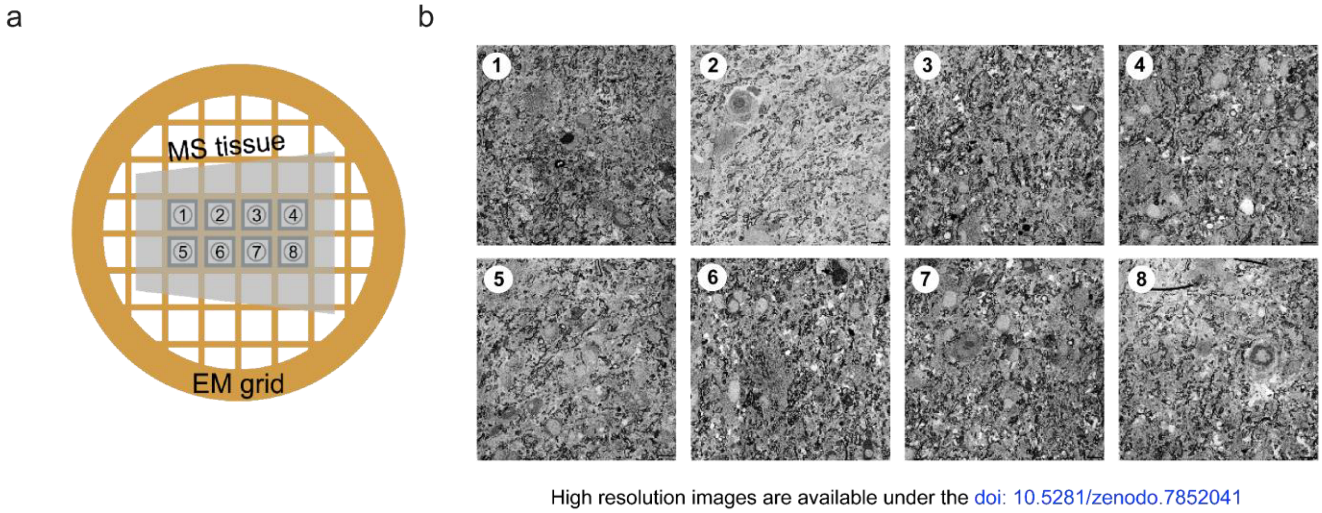

Supplementary Fig. 1: Axonal pathology in human multiple sclerosis. a, Illustration of the imaging strategy to acquire large, high-quality electron micrographs. **b,** Eight large, high-quality electron micrographs of an almost complete sample grid of an MS lesion with moderate demyelination showing typical MS lesion characteristics with immune cell infiltration, phagocytosing cells, myelinated and demyelinated axons as well as the different forms of axonal pathology. The full-resolution images are available as a picture set under the doi: 10.5281/zenodo.7852041. Scale bar = 10μm

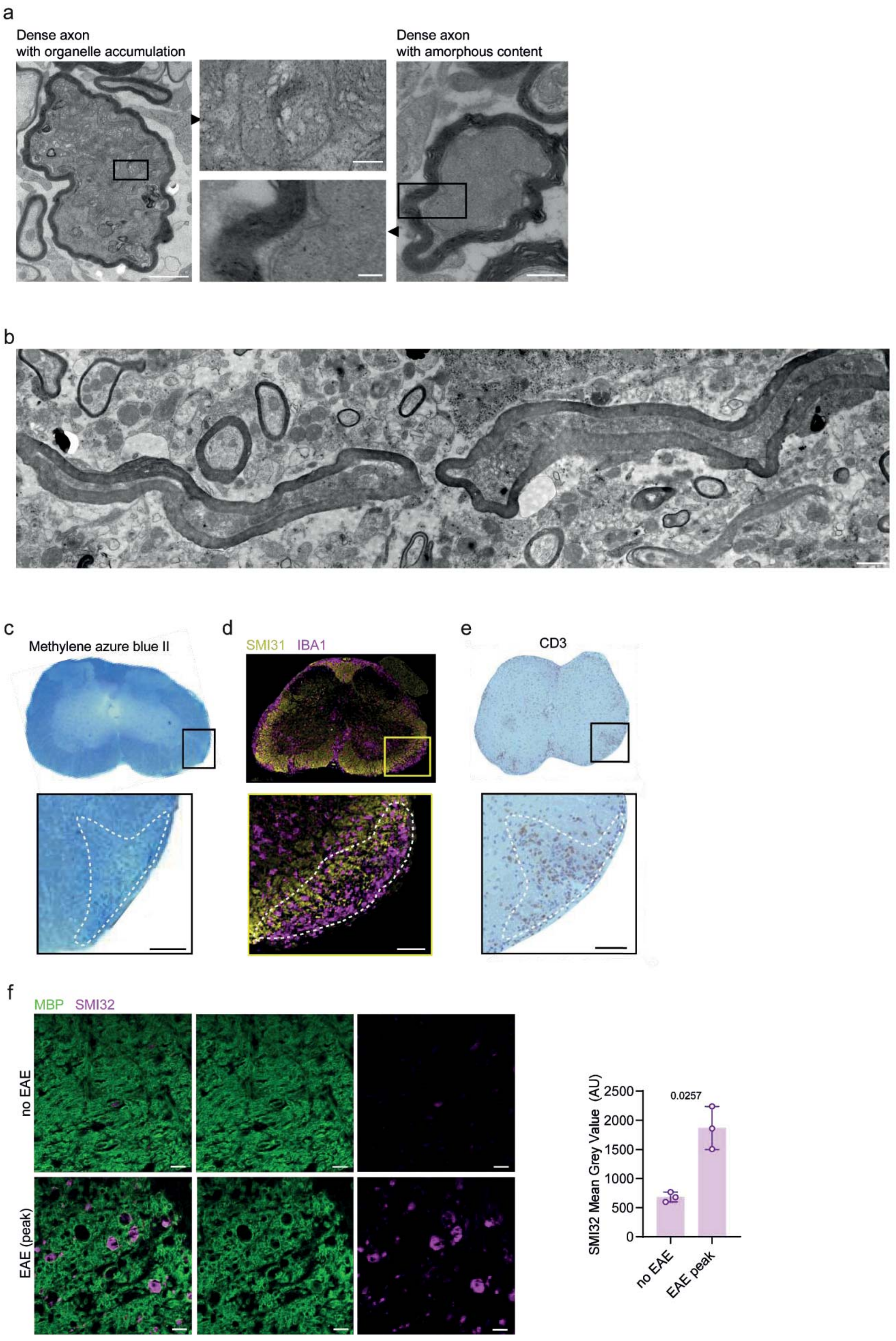
a
Dense axon
with organelle accumulation
Dense axon
with amorphous content
b
c
Methylene azure blue II
d
SMI31 IBA1
e
CD3
f
MBP SMI32
no EAE
EAE (peak)
SMI32 Mean Grey Value (AU)
2500
2000
1500
1000
500
0
0.0257
no EAE
EAE peak

Supplementary Fig. 2: Further characterization of axonal damage types and EAE lesions. a, Representative electron micrographs demonstrating degenerating organelle accumulation (left) and amorphous membranous content (right) in dense axons. Note that the periaxonal space between the myelin sheath and the dense axon can still be seen (center, bottom). Scale bar left and right = 1µm, Scale bar center (zoom-in) = 250nm. **b,** Representative electron micrograph of a longitudinally sectioned dense axon demonstrating that this type of axon pathology extends along the axonal segment. Scale bar = 1µm. **c,** Spinal cord lesions at peak EAE (4d post onset) are identified in methylene azure II-stained semi-thin sections by accumulation of immune cells in the white matter. The white line encircles a lesion. Scale bar = 200µm. **d,** Lesion formation can also be visualized by immunohistochemistry for IBA1, a macrophage/microglia marker and SMI31, a marker for phosphorylated neurofilament. Note the accumulation of $IBA1^+$ cells within the lesion at peak EAE (encircled with a white line). Scale bar = 200µm. **e,** Representative image of a $CD3^+$ staining for T lymphocytes at peak EAE. The area indicated with a white line displays a lesion loaded with CD3+ T cells. Scale bar = 200µm. **f,** (Left) Representative pictures of the ventrolateral spinal cord in healthy wildtype mice compared to peak EAE lesions in the ventrolateral spinal cord immunostained for SMI32 (de-phosphorylated neurofilament, axonal damage marker, pink) and MBP (myelin sheath, green). Scale = 10µm. (Right) Quantification of the mean grey value of the SMI32 signal in the healthy ventrolateral white matter and in peak EAE lesions demonstrates that the intensity of $SMI32^+$ staining is significantly higher in peak EAE lesions. Unpaired two-tailed Welch's t-test. Circles represent biologically independent animals (n=3 per group). Data represents the mean±SD. The p-value is indicated on the graph.

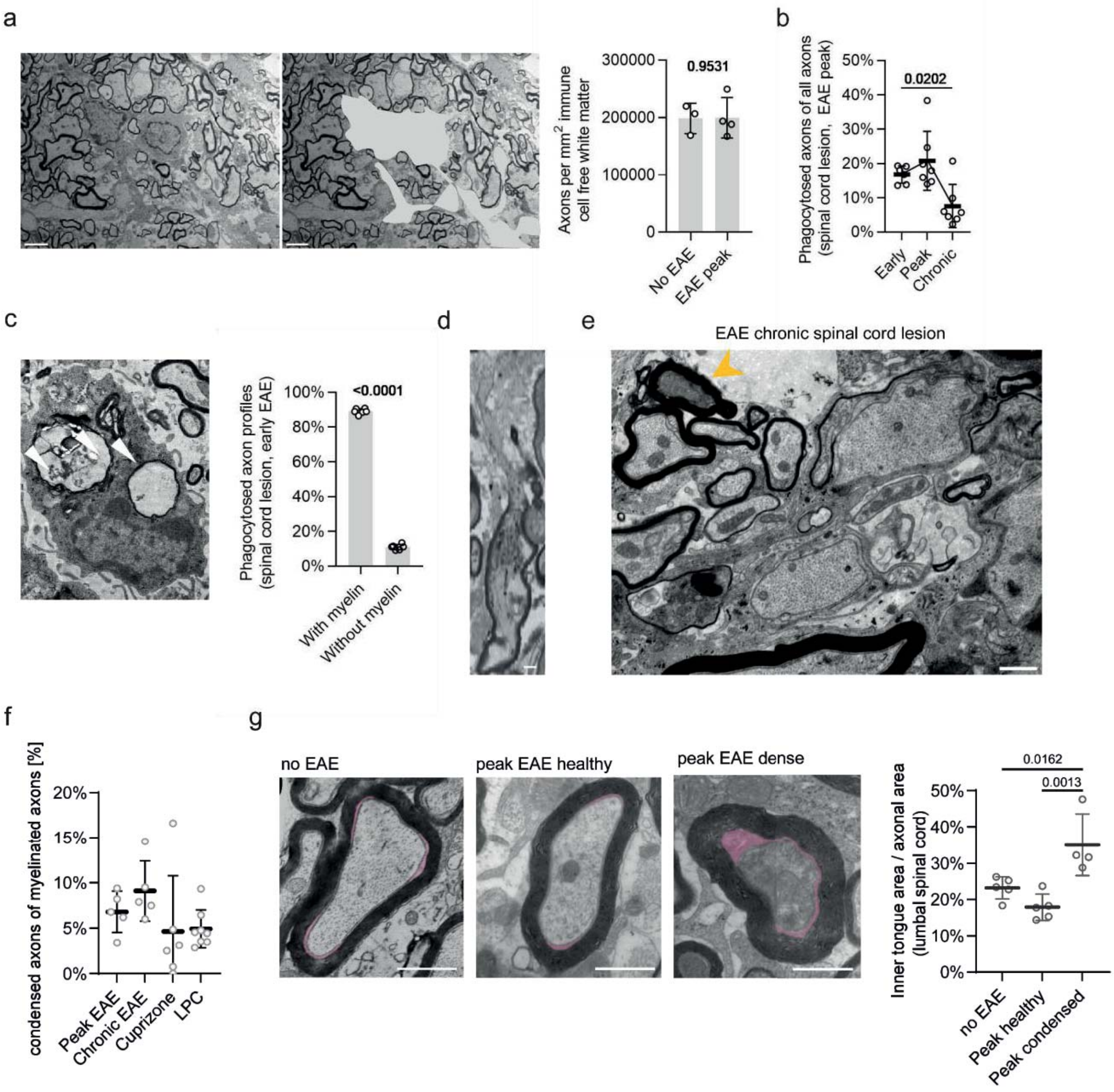

Supplementary Fig. 3: EAE histopathology. a, To estimate axonal numbers in EAE lesions compared to non-EAE controls, the area covered by immune cells was subtracted (right image, grey area) to correct for differences in axonal density due to immune cell occupancy. The respective quantification of axons per mm^2 shows no significant axonal loss at EAE peak (4 days post onset). Scale = 2.5µm. Circles in the graphs represent biologically independent animals (no EAE n=3, EAE peak n=4). **b,** Quantification of the percentage of phagocytosed axons reveals that phagocytosis is most pronounced at EAE peak compared to chronic EAE (40 days post induction). Kruskal-Wallis test with Dunn's multiple comparisons test (p-value for early vs. chronic EAE). Circles in the graphs represent biologically independent animals (Early n=6, Peak n=7, Chronic n=7). **c**, Representative image of a phagocytosing cell at early EAE. Engulfed axon/myelin profiles are indicated by white arrowheads. Quantification of the myelination state of phagocytosed axons at early EAE reveals that most phagocytosed axons

are myelinated. Unpaired two-tailed Welch's t test. Scale bar = 3µm. Circles in the graphs represent biologically independent animals (n=6). **d,** Longitudinal image (Z projection) of the 3D reconstructed axons with organelle accumulations displaying organelle accumulation restricted to the axonal area covered by a myelin sheath. Note that no mechanical constriction is observed at the paranodal area. Scale bar = 1µm. **e,** A representative electron micrograph of a chronic spinal cord EAE lesion shows that demyelinated axons appear relatively intact, while a proportion of myelinated axons show signs of damage like condensation of axoplasm (arrowhead). Scale bar = 1µm. **f,** The percentage of condensed axons in relation to all myelinated axons for peak and chronic EAE, Cuprizone (progressed and full demyelination) and lysolecithin (14d, lesion center and border). Data represents the mean±SD. Circles in the graphs represent biologically independent animals (Peak EAE n=5, Chronic EAE n=5, Cuprizone n=6, LPC n=8). **g,** Representative electron micrographs of a morphologically healthy axon in the unaffected ventrolateral white matter (left), a morphologically healthy axon in a lesion at EAE peak (center) and an axon with condensed cytoplasm in a lesion at EAE peak (right). The inner tongue area is colored in pink. Scale bar = 1µm. Quantification of the inner tongue area in these different axons reveals a specific increase in the area occupied by the inner tongue in axons with condensed cytoplasm at EAE peak. One-way ANOVA with Tukey's post test (n=5 no EAE and peak EAE healthy, n=4 peak EAE dense). Data represents the mean±SD. Circles represent average values of biological replicates. The p-values are written on the graph.

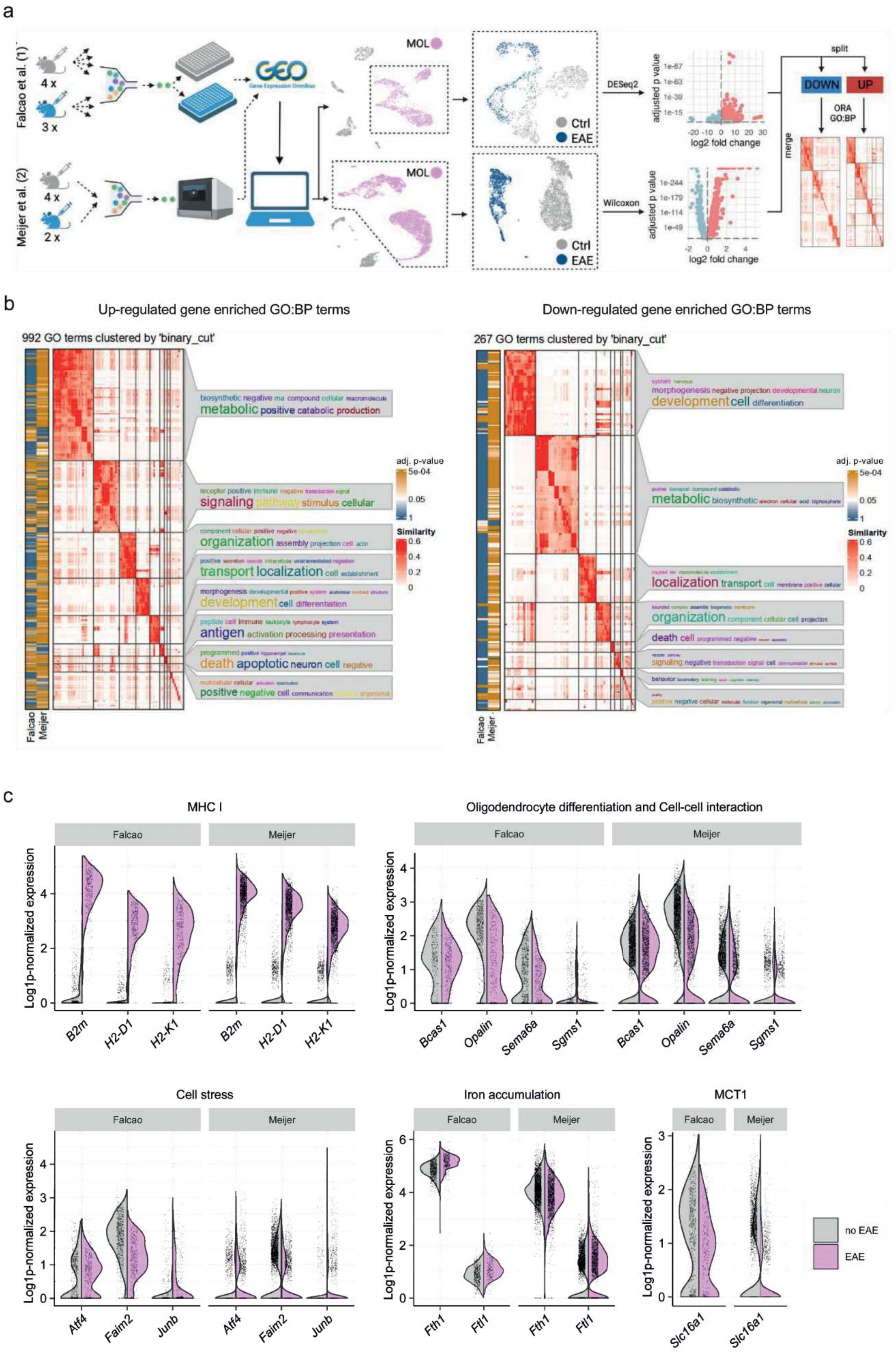
a
Falcao et al. (1)
4 x
3 x
Meijer et al. (2)
4 x
2 x
GEO
Gene Expression Omnibus
MOL
Ctrl
EAE
DESeq2
Wilcoxon
adjusted p value
log2 fold change
split
DOWN
UP
ORA
GO:BP
merge
b
Up-regulated gene enriched GO:BP terms
992 GO terms clustered by 'binary_cut'
Down-regulated gene enriched GO:BP terms
267 GO terms clustered by 'binary_cut'
adj. p-value
5e-04
0.05
1
Similarity
0.6
0.4
0.2
0
Falcao
Meijer
metabolic positive catabolic production
signaling pathway stimulus cellular
organization assembly projection cell actin
transport localization cell establishment
development cell differentiation
antigen activation processing presentation
death apoptotic neuron cell negative
positive negative cell communication
development cell differentiation
metabolic biosynthetic
localization transport
organization
death cell
signaling negative transduction signal cell
c
MHC I
Oligodendrocyte differentiation and Cell-cell interaction
Cell stress
Iron accumulation
MCT1
Falcao
Meijer
Log1p-normalized expression
B2m
H2-D1
H2-K1
Bcas1
Opalin
Sema6a
Sgms1
Atf4
Faim2
Junb
Fth1
Ftl1
Slc16a1
no EAE
EAE

Supplementary Figure 4: Re-analysis of single-cell RNA sequencing data sets on myelinating oligodendrocytes in EAE. a, Schematic representation of the experimental design of the re-analysis of published scRNAseq data (1: Falcão *et al.*, 2018[27] , and 2: Meijer *et al.,* 2022[28]) on myelinating oligodendrocytes in EAE. Mice were injected with MOG to induce EAE or CFA (Ctrl). For (1), GFP+-labeled oligodendrocyte lineage cells (OL) were isolated from individual spinal cords by FACS and sequenced using Smartseq2. For (2), GFP+-labeled OLs were isolated from 4 control and 2 EAE spinal cords, mixed 4:1 with GFP- cells, and sequenced using the 10X Multiome Kit. Processed data (counts) were downloaded, the myelinating oligodendrocytes (MOL) subset extracted according to authors' annotations, and the outliers removed. Differences in gene expression were calculated using DEseq2 (1) or Wilcoxon Rank Sum Test with Bonferroni correction (2). The gene lists were filtered for adjusted p-values < 0.05, split according to positive and negative $\log_2$ fold change and analysed for overrepresentation of Gene Ontology: Biological Process (GO:BP) annotations. **b,** Overrepresentation analysis for GO:BP terms of differentially expressed genes (DEG) for EAE vs Ctrl in MOL from each dataset. Data were split by positive (Up-regulated in EAE, left) and negative (Down-regulated in EAE, right) $\log_2$ fold change. The GO:BP annotations of each gene list are used as input for overrepresentation analysis. The results for both datasets were merged independently for Up and Down. Correlation of GO terms is shown as a heatmap in the middle of each panel. Clusters of terms based on their correlation were derived using the binary cut algorithm (cutoff = 0.85). Keywords for the terms contained in each cluster are displayed on the right of each panel. The p-values for each term in the individual datasets are indicated in the heatmap on the left. **c,** Validation of gene expression in EAE myelinating oligodendrocytes for genes that were reported as dysregulated in myelinating oligodendrocytes in human MS patients[38]. The data are presented in violin plots demonstrating Log1p-normalized gene expression of genes related to MHCI (*B2m, H2-D1, H2-K1*), oligodendrocyte differentiation and cell-cell interaction (*Bcas1, Opalin, Sema6a, Sgms1*), cell stress (*Atf4, Faim2, Junb*), iron accumulation (*Fth1, Ftl1*), in addition to monocarboxylate transporter 1 (*Slc16a1*). Each dot represents a single cell. Data was extracted from Falcão *et al.*, 2018[27] and Meijer *et al.,* 2022[28].

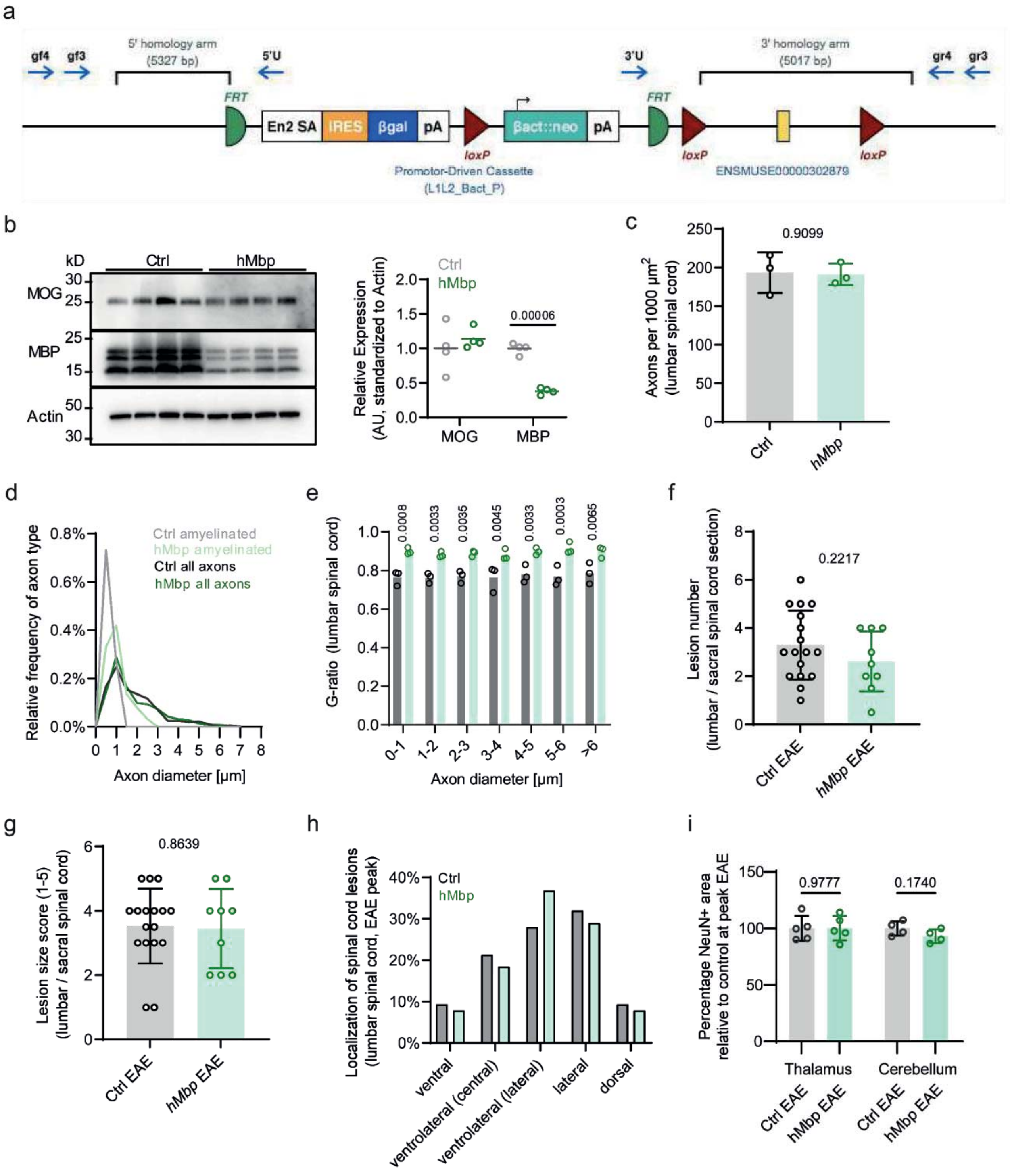

Supplementary Fig. 5: Characterization of *hMbp* mice. a, The genetic construct used in *hMbp* animals leads to a reduced expression of *Mbp* without the necessity to cross-breed with a Cre-line [29]. **b**, Western blot analysis (left) and respective densitometrical quantification (right) of myelin oligodendrocyte protein (MOG), myelin basic protein (MBP) and actin in whole protein lysates from ventrolateral white matter of the lumbosacral spinal cord in wildtype and *hMbp* mice. Quantification demonstrates a strong decrease in the amount of MBP protein but no difference in the amount of MOG protein. N=4 per group, unpaired T test, *P* value given in number. **c,** Quantification of axon numbers in the lumbar spinal cord reveals no difference

between control and *hMbp* mice. Unpaired two-tailed Welch's t test. Circles in the graphs represent biologically independent animals. **d,** Frequency distribution of axonal diameters within the lumbar spinal cord demonstrates similar axon sizes between *hMbp* and controls, and amyelination of medium-sized axons in *hMbp* mice that would normally be myelinated. N = 3 in both groups. **e,** Binned *g*-ratio analysis shows that the hypomyelination of axons in *hMbp* mice is a feature of all axon calibers. N = 3 in both groups, two-way ANOVA with Sidak's post test for each caliber category. **f,g,** Spinal cord EAE lesions occur in similar number and frequency in *hMbp* mice and controls. For each animal, two lumbar sections have been analyzed and the mean is plotted as biological replicate in the diagram. Unpaired two-tailed Welch's t test. Circles in the graphs represent biologically independent animals. **h,** Lesion localization is comparable in both genotypes. In total, 75 lesions of 17 wildtype animals (mean lesions per animal = 4,4) and 38 lesions of 8 *hMbp* animals (mean lesions per animal = 4,2) were quantified. Total lesion numbers are higher than in **g** because lesions can expand to more than one localization. The bars represent the mean distribution of all pooled lesions. **i,** Quantification of the density of $NeuN^+$ neurons (percentage relative to control) in the vermal region of the cerebellum (lobules IV-VI) and the dorsal thalamus. In all diagrams, Circles in the graphs represent biologically independent animals. Data are shown as mean ±SD. Numbers represent p-values.

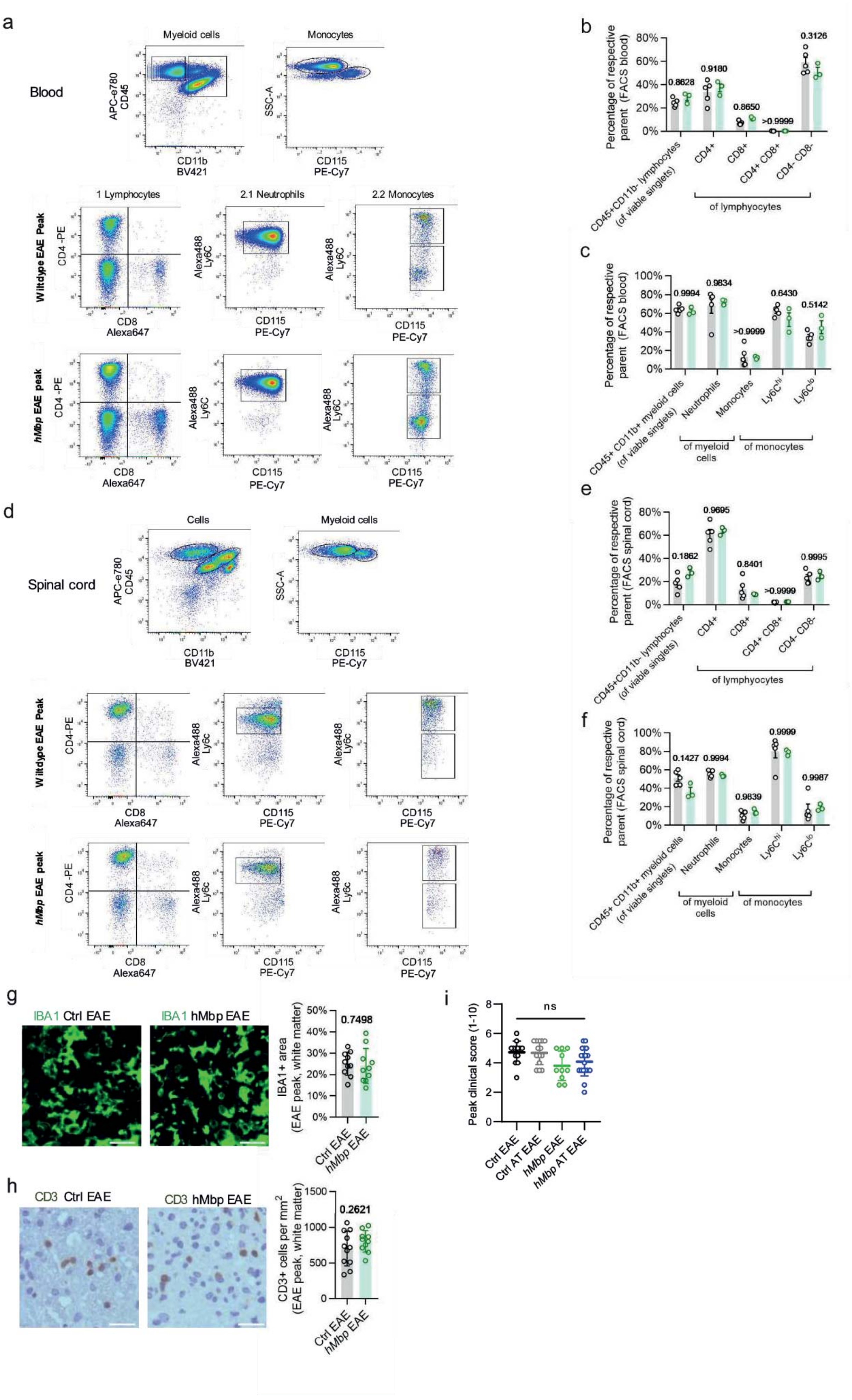
a
Blood
Myeloid cells
Monocytes
APC-e780 CD45
CD11b BV421
SSC-A
CD115 PE-Cy7
1 Lymphocytes
2.1 Neutrophils
2.2 Monocytes
Wiltdype EAE Peak
hMbp EAE peak
CD4 -PE
CD8 Alexa647
Alexa488 Ly6C
CD115 PE-Cy7
b
Percentage of respective parent (FACS blood)
80%
60%
40%
20%
0%
0.8628
0.9180
0.8650
>0.9999
0.3126
CD45+CD11b- lymphocytes (of viable singlets)
CD4+
CD8+
CD4+ CD8+
CD4- CD8-
of lymphyocytes
c
Percentage of respective parent (FACS blood)
100%
80%
60%
40%
20%
0%
0.9994
0.9834
>0.9999
0.6430
0.5142
CD45+ CD11b+ myeloid cells (of viable singlets)
Neutrophils
Monocytes
Ly6Chi
Ly6Clo
of myeloid cells
of monocytes
d
Spinal cord
Cells
Myeloid cells
APC-e780 CD45
CD11b BV421
SSC-A
CD115 PE-Cy7
Wiltdype EAE Peak
hMbp EAE peak
CD4-PE
CD8 Alexa647
Alexa488 Ly6c
CD115 PE-Cy7
e
Percentage of respective parent (FACS spinal cord)
80%
60%
40%
20%
0%
0.1862
0.9695
0.8401
>0.9999
0.9995
CD45+CD11b- lymphocytes (of viable singlets)
CD4+
CD8+
CD4+ CD8+
CD4- CD8-
of lymphyocytes
f
Percentage of respective parent (FACS spinal cord)
100%
80%
60%
40%
20%
0%
0.1427
0.9994
0.9839
0.9999
0.9987
CD45+ CD11b+ myeloid cells (of viable singlets)
Neutrophils
Monocytes
Ly6Chi
Ly6Clo
of myeloid cells
of monocytes
g
IBA1 Ctrl EAE
IBA1 hMbp EAE
IBA1+ area (EAE peak, white matter)
50%
40%
30%
20%
10%
0%
0.7498
Ctrl EAE
hMbp EAE
h
CD3 Ctrl EAE
CD3 hMbp EAE
CD3+ cells per mm2 (EAE peak, white matter)
1500
1000
500
0
0.2621
Ctrl EAE
hMbp EAE
i
Peak clinical score (1-10)
8
6
4
2
0
ns
Ctrl EAE
Ctrl AT EAE
hMbp EAE
hMbp AT EAE

Supplementary Fig. 6: EAE immune cell diversity is similar in control and *hMbp* mice. a, Representative flow cytometry of blood samples at EAE peak (4 dpo). Gate 1 = $CD45^{+}CD11b^{-}$ lymphocytes, gate 2 = $CD45^{+}CD11b^{+}$ myeloid cells (2.1 = $CD45^{+}CD11b^{+}SSc^{hi}CD115^{-}$ neutrophils, 2.2 = $CD45^{+}CD11b^{+}SSc^{lo}CD115^{+}$ monocytes). Ctrl EAE n = 5, *hMbp* n = 3. **b,** Quantification of $CD45^{+}CD11b^{-}CD4^{+}$ and $CD45^{+}CD11b^{-}CD8^{+}$ blood lymphocytes reveals no differences in frequencies between *hMbp* mice and wildtype controls. **c**, The distribution of $CD45^{+}CD11b^{+}$ myeloid cells based on CD115 and Ly6C expression in blood is similar between both genotypes. **d,** Representative gating of FACS spinal cord cells. Gate 1 = $CD45^{+}CD11b^{-}$ lymphocytes, gate 2 = $CD45^{+}CD11b^{+}$ myeloid cells (2.1 = $CD45^{+}CD11b^{+}SSc^{hi}CD115^{-}$ neutrophils, 2.2 = $CD45^{+}CD11b^{+}SSc^{lo}CD115^{+}$ monocytes). **e,f** Quantification of lymphocytes and myeloid cell populations in spinal cord reveals no differences in the frequencies between *hMbp* mice and respective controls. **g,** Representative images of $IBA1^{+}$ cells in the ventrolateral lumbar white matter at EAE peak show no differences in IBA1 density, as also revealed by quantification per area. Unpaired two-tailed Welch's t-test. Scale bar = 20 μm. **h**, Representative images of $CD3^{+}$ cells in the ventrolateral lumbar white matter at EAE peak. The quantification shows similar numbers of $CD3^{+}$ cells in the ventrolateral lumbar white matter. Scale bar = 20 μm. **i,** Comparison of the peak clinical score achieved by each wildtype and *hMbp* mouse either at classical or at adoptive transfer EAE reveals no significant differences between the groups. One-way ANOVA with Tukey's post test. For all graphs, circles represent biologically independent animals. Data are shown as mean ±SD. Numbers represent p-values.

Supplementary Videos

Find supplementary Videos as separate source files in the online version of the article.

Supplementary Video 1: 3D segmentation of an axon with organelle accumulation. This video goes through the cross sections of a 72-μm long electron microscopy block displaying an axon with organelle accumulation. The block was imaged by serial block face scanning electron microscopy (SBF-SEM) at a resolution of 15 nm in XY direction and 240 nm in the Z plane. First, it displays in blue color the segmented myelin sheath, followed by the segmented organelles in pink and the axon in yellow. Scale bar is indicated in the video.

Supplementary Video 2: 3D model of an axon with organelle accumulation. This video displays the model resulting from the segmentation of an axon with organelle accumulations as demonstrated in Suppl. Video 1. The video initially demonstrates each structure separately (myelin in blue, organelles in pink and axon in yellow), and finally displays all of them together as they emerge from the raw cross sections. Importantly, an accumulation of organelles is observed in the myelinated region, while the adjacent amyelinated region appears normal.

Supplementary Video 3: 3D segmentation of an axon with condensed cytoplasm. This video goes through the cross sections of a 72-µm long electron microscopy block displaying an axon with condensed cytoplasm. The block was imaged by serial block face scanning electron microscopy (SBF-SEM) at a resolution of 15 nm in XY direction and 240 nm in the Z plane. First, it displays in blue color the segmented myelin sheath, followed by the axon in yellow.

Supplementary Video 4: 3D model of an axon with condensed cytoplasm. This video displays the model resulting from the segmentation of an axon with condensed cytoplasm as demonstrated in Suppl. Video 3. The video initially demonstrates each structure separately (myelin in blue, axon in yellow), and finally displays all of them together as they emerge from the raw cross sections.

Supplementary Tables

Supplementary Table 1: Table on the characteristics of the four different human MS biopsies.

Biopsy	Age at biopsy	Sex	Biopsy characteristics	Clinical disease characteristics
**L#1**<br>*Near complete demyelination*	28	female	**L:** parietal white matter<br>**EM:** active lesion with immune cell infiltrates, numerous lipid loaden macrophages and near complete demyelination	First manifestation of MS at time of biopsy; no comorbidities
**L#2**<br>*Extensive demyelination*	18	male	**L:** frontal-parietal white matter<br>**EM:** active lesion with immune cell infiltrates, numerous lipid loaden macrophages and extensive demyelination	First manifestation of MS at time of biopsy; no comorbidities
**L#3**<br>*Moderate demyelination*	26	female	**L:** frontal white matter in proximity to the caput nuclei caudati<br>**EM:** active lesion with immune cell infiltrates, lipid loaden macrophages and only moderate extent of demyelination	Relapsing-remitting MS disease course at time of biopsy
**L#4**<br>*Lesion border*	31	male	**L:** frontal white matter<br>**EM:** lesion border with limited/sparse inflammation and demyelination	Relapsing-remitting MS disease course at time of biopsy; secondary chronic progessive disease course

Supplementary Data

Supplementary Data 1: Analysis for overrepresentation of Gene Ontology: Biological Process (GO:BP) annotations in published datasets of the oligodendrocyte response to EAE. Differences in gene expression were calculated using DEseq2 (I) or Wilcoxon's Rank Sum Test with Bonferroni correction (II). Gene lists were filtered for adjusted p values < 0.05, split according to positive and negative log2 fold change and used for over-enrichment analysis with the Gene Ontology: Biological Process (GO:BP) database.

Find supplementary data 1 as separate data file in the online version of the article.
