# Supplementary material for: Myelin insulation as a risk factor for axonal degeneration in autoimmune demyelinating disease
Source: Nat Neurosci. 2023 Jun 29;26(7):1218–28. doi: 10.1038/s41593-023-01366-9 (PMC10322724; doi:10.1038/s41593-023-01366-9)
Supplement: Supplementary file 2 — Reporting Summary [file 41593_2023_1366_MOESM2_ESM.pdf]

nature portfolio

Corresponding author(s): Ruth Martha Stassart
Robert Fledrich
Klaus-Armin Nave

Last updated by author(s): Apr 28, 2023

Reporting Summary

Nature Portfolio wishes to improve the reproducibility of the work that we publish. This form provides structure for consistency and transparency in reporting. For further information on Nature Portfolio policies, see our Editorial Policies and the Editorial Policy Checklist.

Statistics

For all statistical analyses, confirm that the following items are present in the figure legend, table legend, main text, or Methods section.

n/a	Confirmed	
☐	☒	The exact sample size (*n*) for each experimental group/condition, given as a discrete number and unit of measurement
☐	☒	A statement on whether measurements were taken from distinct samples or whether the same sample was measured repeatedly
☐	☒	The statistical test(s) used AND whether they are one- or two-sided<br>*Only common tests should be described solely by name; describe more complex techniques in the Methods section.*
☒	☐	A description of all covariates tested
☐	☒	A description of any assumptions or corrections, such as tests of normality and adjustment for multiple comparisons
☐	☒	A full description of the statistical parameters including central tendency (e.g. means) or other basic estimates (e.g. regression coefficient) AND variation (e.g. standard deviation) or associated estimates of uncertainty (e.g. confidence intervals)
☐	☒	For null hypothesis testing, the test statistic (e.g. *F*, *t*, *r*) with confidence intervals, effect sizes, degrees of freedom and *P* value noted<br>*Give P values as exact values whenever suitable.*
☒	☐	For Bayesian analysis, information on the choice of priors and Markov chain Monte Carlo settings
☒	☐	For hierarchical and complex designs, identification of the appropriate level for tests and full reporting of outcomes
☒	☐	Estimates of effect sizes (e.g. Cohen's *d*, Pearson's *r*), indicating how they were calculated

Our web collection on statistics for biologists contains articles on many of the points above.

Software and code

Policy information about availability of computer code

Data collection	Electron micrographs were aquired using a SIGMA electron microscope (Zeiss) equipped with a STEM detector and ATLAS software<br>Low-magnification chromogenic and fluorescent images, as well as stained semithin sections, were taken with the AxioScan Z1 (Zeiss) using ZEN black.<br>High-magnification fluorescent images were taken with the LSM 880 Airyscan (Zeiss) confocal microscope using ZEN black<br>ACS data were obtained using a FACS Aria III System (BD Biosciences) using FlowJo software.<br>qPCR data were obtained with a qPCR qTOWER84 (Jena Analytics).<br>Single-cell libraries were sequenced using Illumina NextSeq500 and Novaseq 6000.
Data analysis	All 2D images were analysed with FIJI. 3D images were analysed with Microscopy Image Browser (MIB) and IMARIS. All statistics were calculated using GraphPad Prism v7.05. Single cell data was analysed using R v4.1.2 primarily with the help of the package Seurat v.4.1.1. Individual gene lists from re-analysis were used for over-enrichment analysis using GProfiler 2 (v.0.2.1). Significantly enriched GO terms were analysed and visualised for their similarities using the R package simplifyEnrichment (v.1.8.0). The exact code to reproduce figures of the single cell data can be found at https://github.com/nikofleischer/myelin-axonal-damage. Supplementary Data is displayed using Microsoft Office Excel version 2303. ZEISS ATLAS software version 5 was used to acquire 3D electron microscopy images

For manuscripts utilizing custom algorithms or software that are central to the research but not yet described in published literature, software must be made available to editors and reviewers. We strongly encourage code deposition in a community repository (e.g. GitHub). See the Nature Portfolio guidelines for submitting code & software for further information.

Data

Policy information about availability of data

All manuscripts must include a data availability statement. This statement should provide the following information, where applicable:

- Accession codes, unique identifiers, or web links for publicly available datasets
- A description of any restrictions on data availability
- For clinical datasets or third party data, please ensure that the statement adheres to our policy

All relevant data of the present manuscript are available from the corresponding authors on reasonable request. Sequencing data for all mouse samples generated for this study are deposited in the NCBI GEO database (GSE222063, https://www.ncbi.nlm.nih.gov/geo/query/acc.cgi?acc=GSE222063). Seurat objects and Cell Ranger output files are available in the GEO supplement and raw fastq files can be accessed from SRA linked from the GEO records. The expression data we reanalyzed was accessed on GEO as well (GSE113973, https://www.ncbi.nlm.nih.gov/geo/query/acc.cgi?acc=GSE113973, GSE193238, https://www.ncbi.nlm.nih.gov/geo/query/acc.cgi?acc=GSE193238)

Human research participants

Policy information about studies involving human research participants and Sex and Gender in Research.

Reporting on sex and gender	See supplementary table 1
Population characteristics	See supplementary table 1
Recruitment	N.A.
Ethics oversight	Ethics committee of the University Medical Center Göttingen and University Clinic Freiburg

Note that full information on the approval of the study protocol must also be provided in the manuscript.

Field-specific reporting

Please select the one below that is the best fit for your research. If you are not sure, read the appropriate sections before making your selection.

☒ Life sciences ☐ Behavioural & social sciences ☐ Ecological, evolutionary & environmental sciences

For a reference copy of the document with all sections, see nature.com/documents/nr-reporting-summary-flat.pdf

Life sciences study design

All studies must disclose on these points even when the disclosure is negative.

Sample size	Samples sizes were calculated before conducting experiments and calculated using G*Power Version 3.1.7. Adequate Power (1 – beta-error) was defined as ≥ 80% and the alpha error as 5%. All input data necessary to calculate sample sizes were obtained by prior experience with similar experiments and by preliminary experiments.
Data exclusions	No data were excluded, except otherwise stated in the specific methodological section in the manuscript.
Replication	EAE was repeated for the different cohorts (early, peak and chronic) timepoints and showed similar clinical scores until day of sacrification. For lesion quantification, two spinal cord levels (lumbar and sacral) were analysed and combined. All mouse experiments were replicated in at least 3 biologically independent animals. There were no findings that could not be reproduced. For human analyses, the analyses were performed in 4 biologically independent samples, as stated in the methods, the figure legend of Figure 1 and Supplementary Table 1.
Randomization	For animal studies, organisms were allocated randomly to the experimental groups with only considering the determined genotypes. EAE induction was performed in an alternating fashion between the cages of different (blinded) genotypes to ensure that all mice obtain similar MOG solution. For human studies, no grouping and thus no randomization was necessary.
Blinding	All samples were processed in a single-blinded manner.

Reporting for specific materials, systems and methods

We require information from authors about some types of materials, experimental systems and methods used in many studies. Here, indicate whether each material, system or method listed is relevant to your study. If you are not sure if a list item applies to your research, read the appropriate section before selecting a response.

Materials & experimental systems

n/a	Involved in the study	
☐	☒	Antibodies
☒	☐	Eukaryotic cell lines
☒	☐	Palaeontology and archaeology
☐	☒	Animals and other organisms
☒	☐	Clinical data
☒	☐	Dual use research of concern

Methods

n/a	Involved in the study	
☒	☐	ChIP-seq
☐	☒	Flow cytometry
☒	☐	MRI-based neuroimaging

Antibodies

Antibodies used

SMI31 (Biolegend, 801601), IBA1 (Wako, 019-19741), SMI32 (Biolegend 801701), MBP (I,nvitrogen PA1-10008), Synaptophysin (Synaptic Systems 101 203), APP (Merck MAB348), CD3 (Abcam, ab5690), CD11b-BV421 (BioLegend 101236), Ly6C-Alexa488 (BioLegend 128022), CD4-PE (BioLegend 100512), CD8b-Alexa647 (BioLegend 126611), CD45-APC-e780 (Invitrogen, 47-0451-82), CD115-PE-Cy7 (Invitrogen, 25-1152-82), CD4 Pe-Cy5 (Biolegend, GK1.5, 100409), CD3e APC (Biolegend,100311 clone 145-2C11), NEUN (Merck Millipore, MAB377), MOG (Abcam, ab109746), MBP for western blot (Sigma, Atlas, AMAB91064), Beta-Actin (Sigma, A5441), Cy2 goat anti mouse (Dianova, 115-225-071), Cy2 goat anti rat (Dianova, 112-225-143), Cy2 goat anti rabbit (Dianova, 111-225-144), Cy3 goat anti mouse (Dianova, 115-165-071), Cy3 goat anti rat (Dianova, 112-165-003), Cy3 goat anti rabbit (Dianova, 111-165-144), Cy3 goat anti chicken (Dianova, 303-165-003), goat anti mouse (Southern Biotech, 1012-08), goat anti rat (Southern Biotech, 3052-08).

Validation

SMI31 (Biolegend, 801601), Reactivity: Human, Mouse, Rat, 28 citations, IHC-P, WB, ICC
IBA1 (Wako, 019-19741), Reactivity: Human, Mouse, Rat, 24 citations, ICC, IHC
SMI32 (Biolegend 801701), Reactivity: Human, Mouse, Rat, Other mammalian, 9 citations, IHC-P, WB, ICC
MBP (Invitrogen PA1-10008), Reactivity: Bovine, Human, Mouse, Pig, Rat, 1 citation, WB, IHC, ICC/IF
APP (Merck MAB348), Reactivity: Ca, H, M, Mk, Po, R, F, >85 citations, ICC, IF, IHC, IH(P), WB
CD3 (Abcam, ab5690), Reactivity: human, 0 citations, IHC, IHC-P, IHC-Fr, WB, Flo
CD11b-BV421 (BioLegend 101236), Reactivity: Mouse, Human, 61 citations, FC
Ly6C-Alexa488 (BioLegend 128022), Reactivity: Mouse, 25 citations, FC
CD4-PE (BioLegend 100512), Reactivity: Mouse, 33 citations, FC
CD8b-Alexa647 (BioLegend 126611), Reactivity: Mouse, 0 citations, FC, IHC-F
CD45-APC-e780 (Invitrogen, 47-0451-82), Reactivity: Mouse, 61 citations, FC
CD115-PE-Cy7 (Invitrogen, 25-1152-82), Reactivity: Mouse, 10 citations, FC
CD4 Pe-Cy5 (Biolegend, GK1.5), Reactivity: Mouse, 120 citations, FC
CD3e APC (Biolegend,145-2C11), Reactivity: Mouse, 72 citations, FC
NEUN (Merck Millipore, MAB377), Reactivity: Mouse, >1000 citations, FC, IC, IF, IH, IH(P), IP and WB
MOG (Abcam, ab109746), Reactivity: Rabbit, 14 citations, WB, IHC-P
MBP for western blot (Sigma, Atlas, AMAB91064), Reactivity: Mouse, 4 Citations, WB, IHC
Beta-Actin (Sigma, A5441), Reactivity: Mouse, >1000 citations, IHC, ELISA, IF, WB
Cy2 goat anti mouse (Dianova, 115-225-071), Reactivity: Mouse IgG (H+L), 4 citations, IHC
Cy2 goat anti rat (Dianova, 112-225-143), Reactivity: Rat IgG (H+L), 4 citations, IHC
Cy2 goat anti rabbit (Dianova, 111-225-144), Reactivity: Rabbit IgG (H+L), 115 citations, IHC
Cy3 goat anti mouse (Dianova, 115-165-071), Reactivity: Mouse IgG (H+L), 13 citations, IHC
Cy3 goat anti rat (Dianova, 112-165-003), Reactivity: Rat IgG (H+L), 37 citations, IHC
Cy3 goat anti rabbit (Dianova, 111-165-144), Reactivity: Rabbit IgG (H+L), 491 citations, IHC
Cy3 goat anti chicken (Dianova, 303-165-003), Reactivity: Chicken IgY (IgG)(H+L), 17 citations, IHC
goat anti mouse (Southern Biotech, 1012-08), Reactivity: Mouse IgG, 26 citations, IHC, WB, ELISA, Flow cytometry
goat anti rat (Southern Biotech, 3052-08), Reactivity: Rat IgG (H+L), 18 citations, IHC, Flow cytometry, ELISA

Animals and other research organisms

Policy information about studies involving animals; ARRIVE guidelines recommended for reporting animal research, and Sex and Gender in Research

Laboratory animals

Control mice (wildtype) C57BL/6J, 10-14 weeks, all sexes
hMbp mice (MBP<tm1a>/Kan), 10-14 weeks, all sexes
Nur77::GFP+::RFP+ reporter mice (C57BL/6-Tg(Nr4a1-EGFP/cre)820Khog/J), 10-14 weeks, all sexes

Wild animals

This study did not involved wild animals

Reporting on sex

For EAE experiments, both sexes were used.
For lysolecithin and cuprizone experiments, only males were used.

Field-collected samples

This study did not include field-collected samples

Ethics oversight

Niedersächsisches Landesamt für Verbraucherschutz und Lebensmittelsicherheit (LAVES) and MPI of Experimental Medicine, Göttingen; Landesdirektion Sachsen and Paul-Flechsig-Institute, Leipzig

Note that full information on the approval of the study protocol must also be provided in the manuscript.

Flow Cytometry

Plots

Confirm that:

☒ The axis labels state the marker and fluorochrome used (e.g. CD4-FITC).

☒ The axis scales are clearly visible. Include numbers along axes only for bottom left plot of group (a 'group' is an analysis of identical markers).

☒ All plots are contour plots with outliers or pseudocolor plots.

☒ A numerical value for number of cells or percentage (with statistics) is provided.

Methodology

Sample preparation	For standard EAE flow cytometry: Mice were perfused with PBS and heparin after blood was taken from the right ventricle into an EDTA pre-filled tube on ice. The spinal cord was dissected and dissociated with a scalpel on a drop of medium containing Hanks' balance salt solution (HBSS), glucose and HEPES. The cell solution was filtered through a 40 µm strainer, centrifuged, re-suspended in 75% Percoll (GE Healthcare) and layered under a 25% Percoll solution topped with PBS. After centrifugation for 30 minutes with slow breaking, distinct gradients became visible and myelin debris was carefully removed. The remaining cell phase was taken up, cleaned by centrifugation with PBS and stained with viability dye (eFluor 506 Fixable Viability Dye, Thermo Fisher) for 30 minutes on ice. In parallel, erythrocyte lysis using (BD Pharma) was performed for blood samples. For both sample types, cells were blocked with Fc block (CD16/32, Invitrogen, 14-0161-82) for 20 minutes on ice. After washing, antibodies were incubated for 20 minutes on ice.<br>For adoptive transfer flow cytometry: Mice were perfused as described above and the spinal cord digested with Liberase (Roche) and DNAse I (Sigma) for 15 min at 37°C in the water bath. Subsequently, a single cell suspension was prepared and myelin was removed by Percoll gradient (40%) centrifugation. After centrifugation, the cell pellet was re-suspended in FACS buffer and filtered through a 40 µM filter. The cells were blocked using an Fc blocking antibody (Biolegend Fc-block, 2.4G2) and subsequently stained.
Instrument	FACS Aria III System
Software	FlowJo (Tree Star)
Cell population abundance	Cell populations were not collected after sorting.
Gating strategy	Small debris was removed with the preliminary FSC/SSC gate. Single, living cells were obtained by doublet exclusion and exclusion of dead cells using a viability dye. Voltage and gating parameters were set using compensation beads.

☒ Tick this box to confirm that a figure exemplifying the gating strategy is provided in the Supplementary Information.
