# Supplementary material for: Myelin insulation as a risk factor for axonal degeneration in autoimmune demyelinating disease
Source: Nat Neurosci. 2023 Jun 29;26(7):1218–28. doi: 10.1038/s41593-023-01366-9 (PMC10322724; doi:10.1038/s41593-023-01366-9)

Uncropped Western Blot results

This are the uncropped Western Blot results shown in Supplementary Figure 5b.

MOG

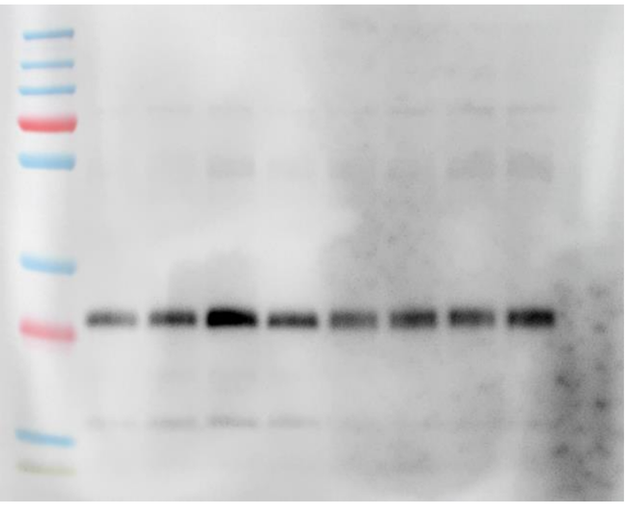

MBP

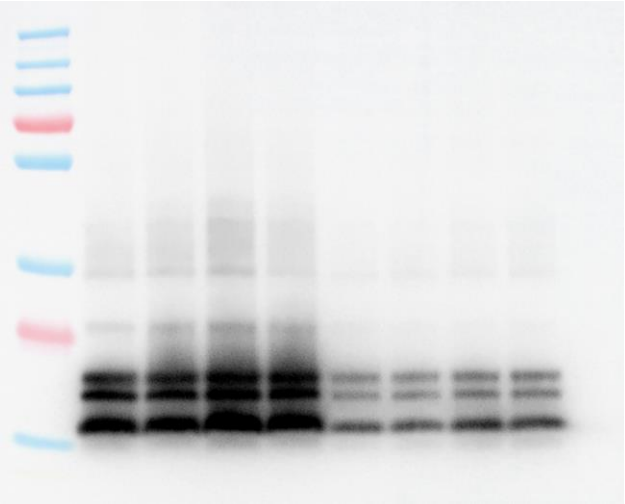

Actin

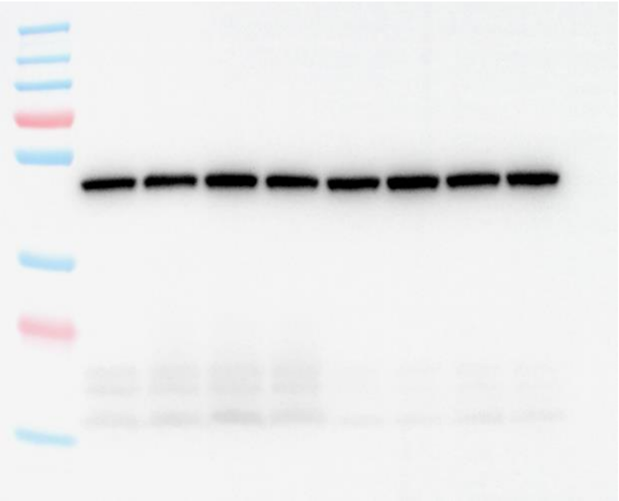

Supplement: Supplementary file 8 — Uncropped Western blots for Supplementary Fig. 5b. [file 41593_2023_1366_MOESM8_ESM.pdf]
